# An unbiased ranking of murine dietary models based on their proximity to human metabolic dysfunction-associated steatotic liver disease (MASLD)

**DOI:** 10.1038/s42255-024-01043-6

**Published:** 2024-06-12

**Authors:** Michele Vacca, Ioannis Kamzolas, Lea Mørch Harder, Fiona Oakley, Christian Trautwein, Maximilian Hatting, Trenton Ross, Barbara Bernardo, Anouk Oldenburger, Sara Toftegaard Hjuler, Iwona Ksiazek, Daniel Lindén, Detlef Schuppan, Sergio Rodriguez-Cuenca, Maria Manuela Tonini, Tamara R. Castañeda, Aimo Kannt, Cecília M. P. Rodrigues, Simon Cockell, Olivier Govaere, Ann K. Daly, Michael Allison, Kristian Honnens de Lichtenberg, Yong Ook Kim, Anna Lindblom, Stephanie Oldham, Anne-Christine Andréasson, Franklin Schlerman, Jonathon Marioneaux, Arun Sanyal, Marta B. Afonso, Ramy Younes, Yuichiro Amano, Scott L. Friedman, Shuang Wang, Dipankar Bhattacharya, Eric Simon, Valérie Paradis, Alastair Burt, Ioanna Maria Grypari, Susan Davies, Ann Driessen, Hiroaki Yashiro, Susanne Pors, Maja Worm Andersen, Michael Feigh, Carla Yunis, Pierre Bedossa, Michelle Stewart, Heather L. Cater, Sara Wells, Jörn M. Schattenberg, Quentin M. Anstee, The LITMUS Investigators, Quentin M. Anstee, Ann K. Daly, Simon Cockell, Dina Tiniakos, Pierre Bedossa, Alastair Burt, Fiona Oakley, Heather J. Cordell, Christopher P. Day, Kristy Wonders, Paolo Missier, Matthew McTeer, Luke Vale, Yemi Oluboyede, Matt Breckons, Jo Boyle, Patrick M. Bossuyt, Hadi Zafarmand, Yasaman Vali, Jenny Lee, Max Nieuwdorp, Adriaan G. Holleboom, Athanasios Angelakis, Joanne Verheij, Vlad Ratziu, Karine Clément, Rafael Patino-Navarrete, Raluca Pais, Valerie Paradis, Detlef Schuppan, Jörn M. Schattenberg, Rambabu Surabattula, Sudha Myneni, Yong Ook Kim, Beate K. Straub, Antonio Vidal-Puig, Michele Vacca, Sergio Rodrigues-Cuenca, Mike Allison, Ioannis Kamzolas, Evangelia Petsalaki, Mark Campbell, Chris J. Lelliott, Susan Davies, Matej Orešič, Tuulia Hyötyläinen, Aidan McGlinchey, Jose M. Mato, Óscar Millet, Jean-François Dufour, Annalisa Berzigotti, Mojgan Masoodi, Naomi F. Lange, Michael Pavlides, Stephen Harrison, Stefan Neubauer, Jeremy Cobbold, Ferenc Mozes, Salma Akhtar, Seliat Olodo-Atitebi, Rajarshi Banerjee, Elizabeth Shumbayawonda, Andrea Dennis, Anneli Andersson, Ioan Wigley, Manuel Romero-Gómez, Emilio Gómez-González, Javier Ampuero, Javier Castell, Rocío Gallego-Durán, Isabel Fernández-Lizaranzu, Rocío Montero-Vallejo, Morten Karsdal, Daniel Guldager Kring Rasmussen, Diana Julie Leeming, Antonia Sinisi, Kishwar Musa, Estelle Sandt, Maria Manuela Tonini, Elisabetta Bugianesi, Chiara Rosso, Angelo Armandi, Fabio Marra, Amalia Gastaldelli, Gianluca Svegliati, Jérôme Boursier, Sven Francque, Luisa Vonghia, An Verrijken, Eveline Dirinck, Ann Driessen, Mattias Ekstedt, Stergios Kechagias, Hannele Yki-Järvinen, Kimmo Porthan, Johanna Arola, Saskia van Mil, George Papatheodoridis, Helena Cortez-Pinto, Ana Paula Silva, Cecilia M. P. Rodrigues, Luca Valenti, Serena Pelusi, Salvatore Petta, Grazia Pennisi, Luca Miele, Antonio Liguori, Andreas Geier, Monika Rau, Christian Trautwein, Johanna Reißing, Guruprasad P. Aithal, Susan Francis, Naaventhan Palaniyappan, Christopher Bradley, Paul Hockings, Moritz Schneider, Philip N. Newsome, Stefan Hübscher, David Wenn, Jeremy Magnanensi, Aldo Trylesinski, Rebeca Mayo, Cristina Alonso, Kevin Duffin, James W. Perfield, Yu Chen, Mark L. Hartman, Carla Yunis, Melissa Miller, Yan Chen, Euan James McLeod, Trenton Ross, Barbara Bernardo, Corinna Schölch, Judith Ertle, Ramy Younes, Harvey Coxson, Eric Simon, Joseph Gogain, Rachel Ostroff, Leigh Alexander, Hannah Biegel, Mette Skalshøi Kjær, Lea Mørch Harder, Naba Al-Sari, Sanne Skovgård Veidal, Anouk Oldenburger, Jens Ellegaard, Maria-Magdalena Balp, Lori Jennings, Miljen Martic, Jürgen Löffler, Douglas Applegate, Richard Torstenson, Daniel Lindén, Céline Fournier-Poizat, Anne Llorca, Michael Kalutkiewicz, Kay Pepin, Richard Ehman, Gerald Horan, Gideon Ho, Dean Tai, Elaine Chng, Teng Xiao, Scott D. Patterson, Andrew Billin, Lynda Doward, James Twiss, Paresh Thakker, Zoltan Derdak, Hiroaki Yashiro, Henrik Landgren, Carolin Lackner, Annette Gouw, Prodromos Hytiroglou, Olivier Govaere, Clifford Brass, Dina Tiniakos, James W. Perfield, Evangelia Petsalaki, Peter Davidsen, Antonio Vidal-Puig

**Affiliations:** 1 TVP Lab, WT/MRC Institute of Metabolic Science, University of Cambridge, Cambridge, UK; 2 Interdisciplinary Department of Medicine, University of Bari “Aldo Moro”, Bari, Italy; 3 Laboratory of Liver Metabolism and MASLD, Roger Williams Institute of Hepatology, London, UK; 4 European Molecular Biology Laboratory, European Bioinformatics Institute (EMBL-EBI), Wellcome Genome Campus, Hinxton, Cambridge, UK; 5 Research and Early Development, Novo Nordisk A/S, Måløv, Copenhagen, Denmark; 6 Newcastle Fibrosis Research Group, Biosciences Institute, Faculty of Medical Sciences, Newcastle University, Newcastle upon Tyne, UK; 7 Department of Medicine III, University Hospital RWTH Aachen, Aachen, Germany; 8 Internal Medicine research Research Unit, Pfizer Worldwide Research and Development, Cambridge, MA USA; 9 CardioMetabolic Diseases Research, Boehringer Ingelheim Pharma GmbH & Co. KG, Biberach an der Riß, Germany; 10 Novartis Institutes for BioMedical Research, Novartis Pharma AG, Basel, Switzerland; 11 Bioscience Metabolism, Research and Early Development Cardiovascular, Renal and Metabolism (CVRM), AstraZeneca BioPharmaceuticals R&D, Gothenburg, Sweden; 12 Division of Endocrinology, Department of Neuroscience and Physiology, Sahlgrenska Academy, University of Gothenburg, Gothenburg, Sweden; 13 Institute of Translational Immunology and Research Center for Immunotherapy, Johannes Gutenberg University Medical Center, Mainz, Germany; 14 Luxembourg Institute of Health, Translational Medicine Operations Hub, Dudelange, Luxembourg; 15 R&D Diabetes & Portfolio Innovation and Excellence, Sanofi-Aventis Deutschland GmbH, Industriepark Hoechst, Frankfurt, Germany; 16 R&D Diabetes, Sanofi-Aventis Deutschland GmbH, Industriepark Hoechst, Frankfurt, Germany; 17 Fraunhofer Institute for Translational Medicine and Pharmacology ITMP, Fraunhofer Innovation Center TheraNova and Goethe University, Frankfurt, Germany; 18 Research Institute for Medicines, Faculty of Pharmacy, Universidade de Lisboa, Lisbon, Portugal; 19 Bioinformatics Support Unit, Faculty of Medical Sciences, Newcastle University, Newcastle upon Tyne, UK; 20 Translational and Clinical Research Institute, Faculty of Medical Sciences, Newcastle University, Newcastle upon Tyne, UK; 21 Liver Unit, Cambridge University Hospitals NHS Foundation Trust & Cambridge NIHR Biomedical Research Centre, Cambridge, UK; 22 Bioscience Metabolism, Research and Early Development Cardiovascular, Renal and Metabolism (CVRM), AstraZeneca BioPharmaceuticals R&D, Gaithersburg, MD USA; 23 Bioscience Cardiovascular, Research and Early Development Cardiovascular, Renal and Metabolism (CVRM), AstraZeneca BioPharmaceuticals R&D, Gothenburg, Sweden; 24 Inflammation and Immunology Research Unit, Pfizer Worldwide Research and Development, Cambridge, MA USA; 25 Fleur De Lis Holdings 10201 Dakins Dr. Richmond, Richmond, VA USA; 26 Department of Internal Medicine, Virginia Commonwealth University, Richmond, VA USA; 27 Boehringer Ingelheim International GmbH, Ingelheim am Rhein, Germany; 28 Research, Takeda Pharmaceutical Company Limited, Fujisawa, Japan; 29 Division of Liver Diseases, Icahn School of Medicine at Mount Sinai, New York, NY USA; 30 Global Computational Biology and Digital Sciences, Boehringer Ingelheim Pharma GmbH & Co. KG, Biberach an der Riß, Germany; 31 Department of Imaging and Pathology, Université Paris Diderot and Hôpital Beaujon, Paris, France; 32 Newcastle NIHR Biomedical Research Centre, Newcastle upon Tyne Hospitals NHS Trust, Newcastle upon Tyne, UK; 33 Department of Pathology, Aretaeion Hospital, Medical School, National and Kapodistrian University of Athens, Athens, Greece; 34 Department of Cellular Pathology, Cambridge University Hospitals NHS Foundation Trust, Cambridge, UK; 35 Department of Pathology, Antwerp University Hospital, Edegem, Belgium; 36 Department of Molecular Imaging, Pathology, Radiotherapy, Oncology. Faculty of Medicine and Health Sciences, University of Antwerp, Wilrijk, Belgium; 37 Research, Takeda Pharmaceuticals Company Limited, Cambridge, MA USA; 38 Gubra, Hoersholm, Denmark; 39 Pfizer, Inc.; Internal Medicine and Hospital, Pfizer Research and Development, Lake Mary, FL USA; 40 LiverPat, Paris, France; 41 Mary Lyon Centre, MRC Harwell, Harwell Campus, Oxford, UK; 42 Department of Internal Medicine II, Saarland University Medical Centre, Homburg, Germany; 43 Lilly Research Laboratories, Eli Lilly and Company, Indianapolis, IN USA; 44 Centro de Investigacion Principe Felipe, Valencia, Spain; 45 Newcastle University, Newcastle, UK; 46 AMC Amsterdam, Amsterdam, Netherlands; 47 Institute of Cardiometabolism And Nutrition, Paris, France; 48 Hôpital Beaujon, Assistance Publique Hopitaux de Paris, Paris, France; 49 University Medical Center Mainz, Mainz, Germany; 50 University of Cambridge, Cambridge, UK; 51 Örebro University, Örebro, Sweden; 52 Center for Cooperative Research in Biosciences, Derio, Spain; 53 University of Bern, Bern, Switzerland; 54 University of Oxford, Oxford, UK; 55 Perspectum, Oxford, UK; 56 Servicio Andaluz de Salud, Seville, Spain; 57 Nordic Bioscience, Herlev, Denmark; 58 Integrated Biobank of, Luxembourg, Luxembourg; 59 University of Torino, Torino, Italy; 60 Università degli Studi di Firenze, Florence, Italy; 61 Consiglio Nazionale delle Ricerche, Rome, Italy; 62 Università Politecnica delle Marche, Ancona, Italy; 63 University Hospital of Angers, Angers, France; 64 Antwerp University Hospital, Antwerp, Belgium; 65 Linköping University, Linköping, Sweden; 66 University of Helsinki, Helsinki, Finland; 67 UMC Utrecht, Utrecht, Netherlands; 68 Medical School of National & Kapodistrian University of Athens, Athens, Greece; 69 Faculdade de Medicina, Universidade de Lisboa, Centro Hospitalar Vila Nova de Gaia, Lisbon, Portugal; 70 Faculty of Pharmacy, Universidade de Lisboa, Lisbon, Portugal; 71 Università degli Studi di Milano, Milan, Italy; 72 Università degli Studi di Palermo, Palermo, Italy; 73 Università Cattolica del Sacro Cuore, Rome, Italy; 74 University Hospital Würzburg, Würzburg, Germany; 75 RWTH Aachen University Hospital, Aachen, Germany; 76 University of Nottingham, Nottingham, UK; 77 Antaros Medical, Gothenburg, Sweden; 78 National Institute for Health Research, Biomedical Research Centre at University Hospitals Birmingham NHS Foundation Trust and the University of Birmingham, Birmingham, UK; 79 iXscient, London, UK; 80 Genfit, Lille, France; 81 Intercept Pharma, New York, NY USA; 82 OWL, Coimbra, Portugal; 83 Eli Lilly and Company, Indianapolis, USA; 84 Pfizer, New York, NY USA; 85 Boehringer-Ingelheim, Ingelheim am Rhein, Germany; 86 Somalogic, Boulder, CO USA; 87 Novo Nordisk, Copenhagen, Denmark; 88 Ellegaard Göttingen Minipigs, Dalmose, Denmark; 89 Novartis Pharma AG, Basel, Switzerland; 90 AstraZeneca, Gothenburg, Sweden; 91 Echosens, Paris, France; 92 Resoundant, Rochester, USA; 93 Bristol-Myers Squibb, New York, NY USA; 94 HistoIndex, Singapore, Singapore; 95 Gilead, Foster City, CA USA; 96 RTI-HS, Research Triangle Park, NC USA; 97 Takeda Pharmaceuticals Company Ltd, Osaka, Japan; 98 AbbVie, North Chicago, USA; 99 Medical University of Graz, Graz, Austria; 100 University of Groningen, Groningen, Netherlands; 101 Aristotle University of Thessaloniki, Thessaloniki, Greece; 102 KU Leuven, Leuven, Belgium; 103 Resolution Therapeutics, Paris, France; 104 Present Address: Ferring Pharmaceuticals A/S, International PharmaScience Center, Copenhagen, Denmark

**Keywords:** Non-alcoholic fatty liver disease, Metabolic diseases, Experimental models of disease, Translational research

## Abstract

Metabolic dysfunction-associated steatotic liver disease (MASLD), previously known as non-alcoholic fatty liver disease, encompasses steatosis and metabolic dysfunction-associated steatohepatitis (MASH), leading to cirrhosis and hepatocellular carcinoma. Preclinical MASLD research is mainly performed in rodents; however, the model that best recapitulates human disease is yet to be defined. We conducted a wide-ranging retrospective review (metabolic phenotype, liver histopathology, transcriptome benchmarked against humans) of murine models (mostly male) and ranked them using an unbiased MASLD ‘human proximity score’ to define their metabolic relevance and ability to induce MASH-fibrosis. Here, we show that Western diets align closely with human MASH; high cholesterol content, extended study duration and/or genetic manipulation of disease-promoting pathways are required to intensify liver damage and accelerate significant (F2+) fibrosis development. Choline-deficient models rapidly induce MASH-fibrosis while showing relatively poor translatability. Our ranking of commonly used MASLD models, based on their proximity to human MASLD, helps with the selection of appropriate in vivo models to accelerate preclinical research.

## Abstract (web summary)

The LITMUS consortium provides a resource of rodent MASLD models benchmarked against metabolic, histologic and transcriptomic features that are relevant for human MASLD. The work is useful for selecting relevant rodent models for studying this common disease.

## Main

Non-alcoholic fatty liver disease (NAFLD) is the hepatic manifestation of the metabolic syndrome^1^. It clusters with obesity, insulin resistance, diabetes, dyslipidemia, atherosclerosis and cancer^2,3^. Given its metabolic context, a multi-society consensus statement on fatty liver disease proposed the new nomenclature, metabolic dysfunction-associated steatotic liver disease (MASLD)^4^.

The MASLD spectrum ranges from simple steatosis to steatohepatitis (NASH/MASH) and MASH-fibrosis^5,6^. Under the pressure of environmental factors (lifestyle, nutrition, microbiome) and genetic predisposition (for example, the rs738409 C>G polymorphism in the *PNPLA3* gene), the disease can progress to MASH, promoted by lipotoxic insults driving hepatocyte injury, inflammation and chronic activation of wound-healing responses^7,8^. Progressive MASH places patients at risk of cirrhosis and hepatocellular carcinoma, which may result in liver-related mortality and the need for liver transplantation^1,9^.

The lack of a standard translationally relevant preclinical model has hindered the field’s ability to study the chronic and complex pathophysiology of MASH. Furthermore, overinflated preclinical efficacy data generated from models in which human pathophysiology is not accurately replicated probably contribute to negative clinical trial results in the MASH field. Many diets differing in macronutrient composition have been tested in a wide range of rodent models aiming to feature the whole spectrum of metabolic disease and/or hepatic damage^10–12^. Disappointingly, within each diet macro-category, relatively subtle differences in micronutrient and macronutrient composition (for example, amount of cholesterol or choline) and study designs are sufficient to introduce variability in MASLD disease endpoints^12–16^. Several stressors have been used to accelerate disease progression and homogenize phenotypes, including genetically modified mice and rats (featuring obesity^17^ or hypercholesterolemia^18,19^), chemically induced reduction of insulin secretory capacity^20^ and/or the addition of toxic chemicals (for example, carbon tetrachloride, CCl_4_ (ref. ^21^)), and the use of genetically modified mice that spontaneously develop progressive steatohepatitis (for example, NEMO, PTEN knockout (KO) mice^16,22^). Moreover, some rodent models seem to recapitulate the metabolic aspects of MASLD, whereas others are better at developing its fibro-inflammatory features^12,13,23^. Given the vast proliferation of models, variability and lack of standardization^12–16^, a systematic comparison validating metabolic features, histology and transcriptomics against human disease is warranted but currently lacking.

To resolve the uncertainty about the most relevant preclinical mouse models, we have performed a wide-ranging retrospective analysis of the most common murine models used in academia and the pharmaceutical industry that were available to our consortium and collaborators, benchmarked against human MASLD and ranked according to the following characteristics: (1) obesity and/or metabolic syndrome; (2) development of steatohepatitis with progressive fibrosis (following hard outcomes of clinical trials defined for an amelioration, or at least a non-worsening, of fibrosis^24^); and (3) similarity of the histological features and molecular events to human MASH.

**Fig. 1:**
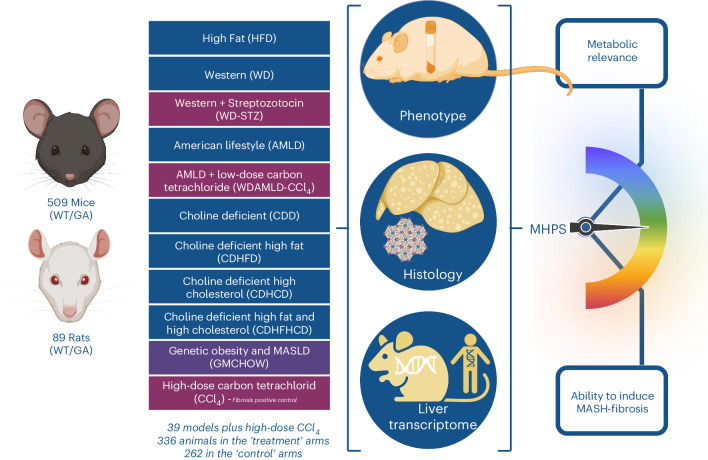
Study design. In this study, we collected retrospective information from 598 animals (509 WT/GA mice, 89 WT/GA rats): 336 animals subjected to treatment (MASLD-inducing conditions: 315 animals; or CCl_4_: 21 animals) and 262 animals as controls for MASLD-inducing conditions (247 animals) or CCl_4_ (15 animals), returning 39 models (that is, study designs) aimed at modeling MASLD, and two time points for CCl_4_ (positive controls of MASLD-independent fibrosis). Details of the study designs (numerosity, species, background, genetic manipulation, diet, time point and room temperature) are provided in Supplementary Table 1. For all the studies, phenotypic information (Supplementary Table 5), centralized histopathology assessment (Supplementary Table 6) and liver transcriptomics (Supplementary Table 4) were available. These data were integrated into an unbiased binary score (MHPS) ranking the models in terms of their metabolic relevance and ability to induce MASH-fibrosis. Created with BioRender (agreement number GG26BHMS6Y).

We developed a bioinformatic pipeline integrating metabolic phenotype data, liver histology (with centralized staining and assessment) and liver transcriptome benchmarked against human disease to create a MASLD ‘human proximity score’ (MHPS; Fig. 1). The MHPS was invaluable in generating a dual ranking of models based on their metabolic relevance and/or ability to induce MASH-fibrosis, highlighting the models that more closely resembled the metabolic and/or the fibrotic features of the disease. Our approach identified murine MASLD models showing (phenotypic and/or histologic and/or transcriptomic) profiles relevant to human MASH, which are suitable for most preclinical experimental applications.

## Results

### Main phenotypic and histologic attributes characterizing human MASLD in murine models

**Fig. 2:**
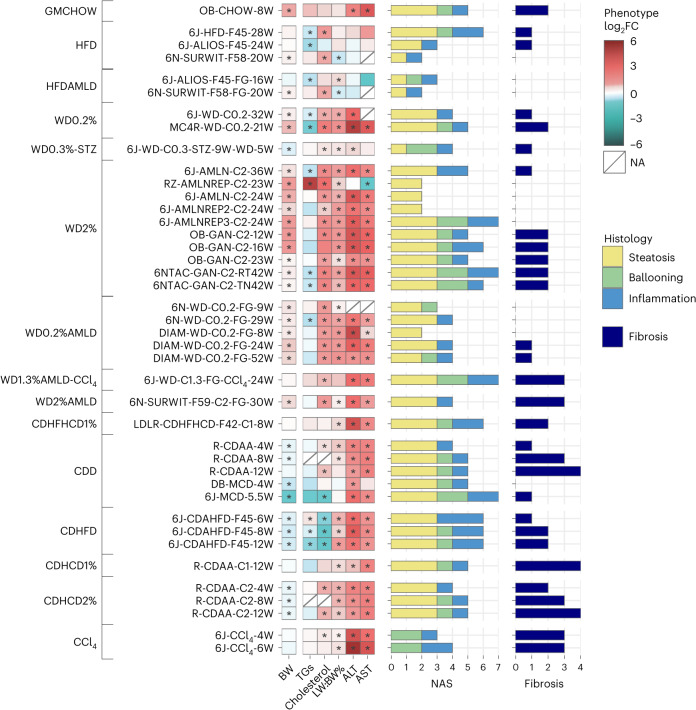
Phenotypic and histologic characterization of the models. Phenotypic changes observed in the MASLD models compared to their matched controls were profiled as the log_2_ fold change (log_2_FC) across measures of BW, blood triglycerides (TGs) and cholesterol, LW:BW% ratio, and ALT and AST. The red–blue color gradient indicates the level of increase–decrease of the measure in the MASLD models compared to their controls, while an asterisk indicates a significant change at *P* < 0.05 (two-sided Mann–Whitney *U*-test). The two panels of horizontal bars give an overview of the complete histological profiles, in which the total length indicates the activity score (CRN NAS) and fibrosis^25^. In addition, NAS components (steatosis, ballooning and inflammation) are represented by the stacked bar (yellow, green and blue, respectively) lengths. All models are grouped according to their macro-categories (detailed by the leftmost annotations). Source data

We collated retrospective data and samples from 39 commonly used murine genetic or dietary MASLD models (treatment: 315 animals; control diet: 247 animals; see Fig. 2 and Supplementary Tables 1 and 2 for more details) available to the consortium and/or collaborators, and clustered them according to macro-categories (diet and/or genetic background) as follows: (1) genetically modified models of obesity or MASLD; (2) high-fat diet (HFD); (3) Western (that is, atherogenic) diet (WD; HFD enriched in refined carbohydrates and cholesterol); these models were subclustered according to cholesterol concentration (0–2%) and/or the use of chemicals (streptozotocin, STZ); (4) American lifestyle diet (AMLD) including HFD (HFDAMLD) or WD (WDAMLD) supplemented with refined sugars in the drinking water, with or without the use of low-dose CCl_4_; and (5) choline-deficient dietary models including ‘canonical’ choline-deficient diets (CDD) and choline-deficient diets supplemented with fat (CDHFD), cholesterol (CDHCD; 1% or 2%) or both (CDHFHCD). (6) As positive controls for isolated fibrosis, we used CCl_4_-treated mice (CCl_4_ treatment: 21 animals; control treatment: 15 animals; two time points).

The anticipated outcome of a MASLD murine model exhibiting body weight (BW) gain was readily achieved in genetically modified (leptin-deficient (ob/ob)) mice, WD and HFD models. Notably, the weight gain was less prominent in HFD models, and in WD or WDAMLD supplemented with chemicals (CCl_4_ or STZ). By contrast, choline-deficient models generally reduced BW with the exception of CDHFHCD1% in low-density lipoprotein receptor (LDLR) KO mice. Most models did not significantly elevate circulating triglyceride levels, except for ZSF1 (diabetic) rats on a WD2% diet (RZ-AMLNREP-C2-23W) and a CDHFD mouse model. As expected, most HFD models and all diets with increased cholesterol content (0.2–2%) (including WD, WDAMLD, CDHCD and CDHFHCD) as well as wild-type rats treated with CDAA exhibited hypercholesterolemia.

Apart from HFDs (and HFDAMLD), most experimental designs resulted in notable liver enlargement (that is, the ratio of liver weight to BW (LW:BW%)) and elevated aspartate aminotransferase (AST) and alanine aminotransferase (ALT) levels, especially at the final time point studied. Data on glucose metabolism was limited to a subset of our models (Supplementary Table 1). However, most HFD and WD diets examined have been documented in the literature to decrease insulin tolerance and impair glucose metabolism; conversely, choline-deficient models are not as well-characterized in this context (see Supplementary Table 2 for details and references).

To assess the histological characteristics of MASLD, the LITMUS (‘Liver Investigation: Testing Marker Utility in Steatohepatitis’ Consortium) Histopathology Group evaluated liver pathology using the Clinical Research Network (CRN)^25^, and Steatosis, Activity and Fibrosis (SAF)^26^ grading and staging systems. This evaluation was conducted on tissue sections centrally stained with hematoxylin and eosin (H&E) and Sirius Red. The NAFLD activity score (NAS) grading confirmed that most models induced moderate to severe steatosis (score 2–3) and mild to moderate lobular inflammation (score 1–2). Analysis of the HFD and HFDAMLD models showed relatively mild MASH activity (characterized by lobular inflammation and hepatocyte ballooning) and fibrosis, resulting in mild steatohepatitis^12,13^. Ballooning (score 1–2) was a prominent feature in choline-deficient models (CDD, CDHFD, CDHCD and CDHFHCD) and some WD models, especially those containing 2% cholesterol (WD2%); for instance, Gubra Amylin Diet (GAN), Amylin Liver NASH (AMLN-C2) or AMLN Replacement Diet 3 (AMLNREP3-C2). Cholesterol at relatively low concentrations (WD0.2%) induced ballooning primarily on genetically modified obese mice (for example, MC4R KO mice), when supplemented with sugar water (WD0.2%AMLD) in both C57BL/6N and Diamond mice, or in those models additionally challenged with chemicals (STZ or CCl_4_).

The highest NAS (score 6–7) was attained in models consuming diets containing 40–45% fat. This was observed across various macro-categories, including HFD-F45, AMLNREP3-C2, GAN-C2, CDAHFD-F45, the WD1.3%AMLD-CCl_4_ model and CDHFHCD1% administered to LDLR KO mice, as well as in the methionine- and choline-deficient diet (MCD) model.

Notably, significant (F2 or higher) fibrosis was prevalent in choline-deficient dietary models (CDD, CDHFD, CDHCD and CDHFHCD1%). By contrast, fibrosis was absent in short-term MCD, indicating that a minimum duration of 8 weeks is necessary for MCD to induce MASH-associated fibrosis. Most animals fed with standard chow, HFD, HFDAMLD, WD or WDAMLD did not develop significant (F2 or higher) fibrosis even after prolonged challenges (lasting 36–52 weeks). However, significant fibrosis was observed in C57BL6/N mice on a WD2%AMLD diet (6N-SURWIT-F59-C2-FG-30W), C57BL/6NTac or ob/ob mice fed the GAN-C2 diet, WD1.3%AMLD-CCl_4_ and MC4R KO mice on a WD0.2% diet.

Our findings suggest that no single model currently replicates all the phenotypic and histological characteristics of human MASLD among the models we evaluated. The HFD, WD and AMLD models, with or without chemical supplementation, are broadly effective in simulating the metabolic aspects of MASLD. However, these diets typically result in a milder histological phenotype, with some notable exceptions. Conversely, the choline-deficient dietary models (CDD, CDHFD, CDHCD and CDHFHCD) rapidly (within 12 weeks or less) lead to the development of MASH with significant fibrosis. However, they fall short in accurately modeling the metabolic burden of MASLD owing to BW loss and improved dyslipidemia. Exceptions to this trend include CDHFHCD-fed LDLR KO mice and certain CDHCD-fed rat models.

### Murine MASLD transcriptomes are close to humans but do not predict fibrosis efficiently

**Fig. 3:**
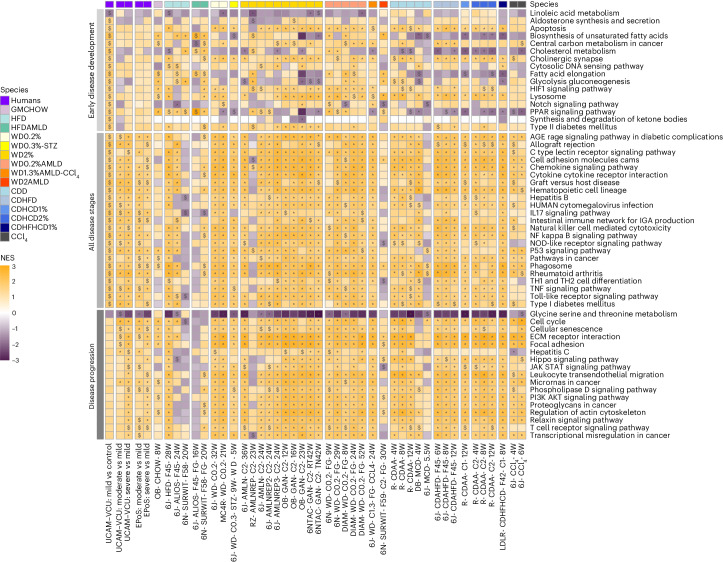
DRPs in human and murine MASLD. Selection of KEGG-affected pathways characterizing the ‘early disease development’, ‘all disease stages’ and ‘disease progression’ groups. The ‘early disease development’ group includes statistically significant (*P* < 0.05) modulated pathways in ‘mild vs controls’ but not in ‘moderate–severe vs mild’ human disease stages comparisons. The ‘all disease stages’ group includes statistically significant and homogeneously modulated pathways at all human disease stages (‘mild vs controls’ and ‘moderate–severe vs mild’ in both UCAM–VCU and EPoS); the ‘disease progression’ group includes homogeneously modulated and statistically significant pathways in ‘moderate–severe vs mild’ comparisons in both UCAM–VCU and EPoS but not in ‘mild vs controls’ comparisons. FGSEA calculated a normalized enrichment score (NES; the enrichment score normalized to mean enrichment of random samples of the same size) for each pathway and determined statistical significance using permutation testing (two-sided), adjusting for multiple comparisons to control the false discovery rate (FDR). The human and murine datasets are represented in a color-scale matrix showing the NES. * and $ symbols denote statistical significance ($, *P* < 0.05; *, FDR < 0.05). All models are grouped according to their macro-categories, as indicated by the panel on the top of the heatmap. Source data

**Extended Data Fig. 1:**
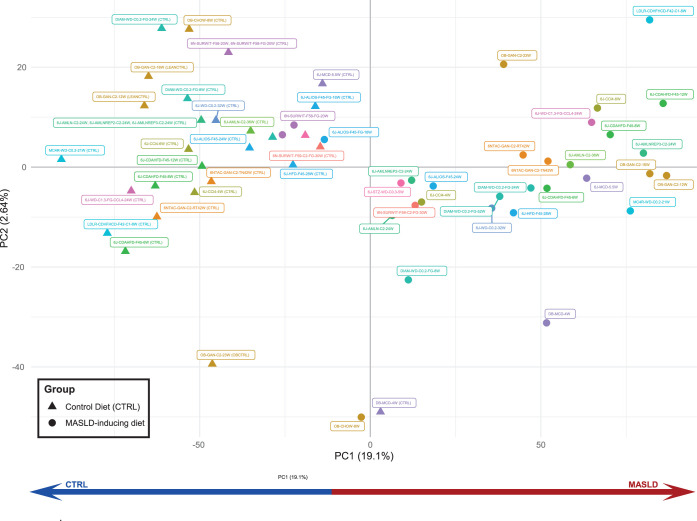
PCA plot of RNASeq data comparing the different mouse MASLD models. The plot uses the normalised batch-corrected gene expression data. Each point represents a mouse model. The Controls (triangles) are clearly separated from the MASLD models (circles) according to the first principal component (PC1). Source data

**Extended Data Fig. 2:**
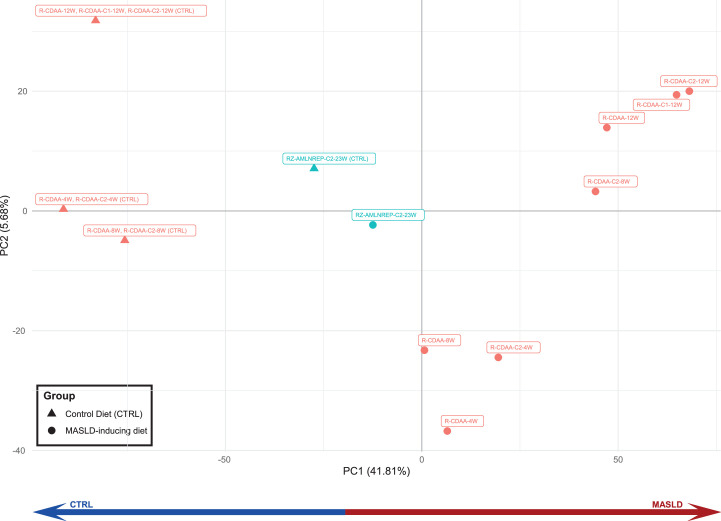
PCA plot of RNASeq data comparing the different rat MASLD models. The plot uses the normalised batch-corrected gene expression data. Each point represents a rat model. The Controls (triangles) are separated from the MASLD models (circles) according to the first principal component (PC1). Source data

Using publicly accessible human NAFLD/MASLD transcriptomes described by our teams^27–29^, we collated two datasets comprising 136 (University of Cambridge (UCAM) and Virginia Commonwealth University (VCU)) and 168 (Newcastle University (EPoS)) patients, as detailed in Supplementary Table 3. We aimed to identify differentially expressed genes (DEGs) and differentially regulated pathways (DRPs) that define MASLD. We identified specific pathways (DRPs; illustrated in Fig. 3) and DEGs (listed in Supplementary Table 4) that are prevalent in the early stages of the disease (that is, mild MASLD vs control), throughout all stages of the disease (that is, across all comparisons within the two datasets) and during the progression of fibrosis (that is, moderate–severe vs mild MASLD). These human datasets served as benchmarks for comparing changes in murine transcriptomics, with the replicability of these findings across various datasets detailed in Supplementary Fig. 1. In our analysis, we observed that diets inducing MASLD formed distinct clusters from control diets when analyzed through the first principal component in mice (Extended Data Figure 1) and in rats (Extended Data Figure 2). Additionally, genetically obese mouse models (ob/ob; leptin receptor-deficient (db/db)) were differentiated from other data on the second principal component, as depicted in Extended Data Figure 1.

**Fig. 4:**
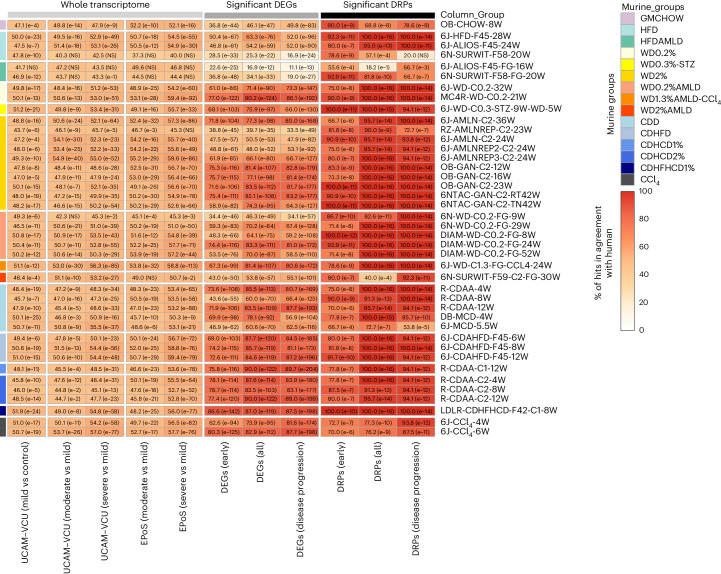
Agreement of murine DEGs and DRPs with human MASLD. Heatmap showing the agreement between murine MASLD models and human data based on the list of significant DEGs, DRPs, or the whole transcriptome. The percentage (%) of agreement between murine and human datasets defines the proportion of DEGs and DRPs statistically modulated and in the same direction compared to the human reference datasets (defined for ‘early disease development’, ‘all disease stages’ and ‘disease progression’ comparisons) or the proportion of DEGs statistically modulated and in the same direction compared to the human disease stage comparisons (defined for the whole transcriptome). All models are grouped according to their macro-categories as indicated in the graphic legend of the figure. Data are represented in a color-scale matrix showing the percentage of agreement and refer to DEGs (Supplementary Table 4; whole dataset or genes defined for ‘early disease development’, ‘all disease stages’ and ‘disease progression’, respectively) and DRPs (Fig. 3). In parenthesis, we show the results of the hypergeometric test (one-sided) performed on the same comparison groups, indicating the statistical significance (NS, non-significant (*P* > 0.05)). Source data

The murine models were compared to human NAFLD/MASLD transcriptomic data to evaluate their alignment in terms of DEGs and DRPs, as illustrated in Fig. 4. At the whole transcriptome level, the comparison of DEGs between murine models and human data showed relatively modest alignment rates (approximately 50%) when considering only fold changes and the direction of regulation for all models. However, when focusing on statistically significant findings in humans (DEGs and DRPs) characterizing ‘early disease development’, ‘all disease stages’ and ‘disease progression’ (as detailed in the Methods), we observed an improved and statistically significant agreement for the majority of murine models. It is important to note that although variable responses were at the individual transcript level among different models, this variability was not mirrored in the DRPs analysis. The DRPs (shown in Fig. 3) demonstrated a high level of agreement with human data, with only a few exceptions, as detailed in Fig. 4.

This analysis indicates that despite variations in specific single gene expression, the biological pathways implicated in the disease were consistently regulated across both the animal models and human cases. Generally, the pathways that characterize ‘all disease stages’ (which include wound healing pathways such as inflammation and cell proliferation) and those that define MASH ‘disease progression’ (which include wound healing pathways such as stellate cell activation, fibrosis, and carcinogenesis) were regulated similarly in humans and in most murine models. Of relevance, significant transcriptional changes associated with ‘disease progression’ in humans were also noted in murine models that paradoxically only exhibited mild (F0–F1) fibrosis. These findings underscore the critical importance of conducting liver histopathology in rodents. Relying solely on transcriptomic data is inadequate, as it does not fully capture the complex pathophysiology of fibrosis or accurately predict extracellular matrix deposition.

Similar to human cases, most murine models displayed significant regulation in the pathways associated with ‘early disease development’, which are involved in the pathophysiology of type 2 diabetes, hypoxia (HIF1 signaling) and lysosome function. However, there were notable species-specific differences in regulating cholesterol and lipid metabolism pathways among different macro-clusters, as detailed in Figs. 3 and 4. HFD and WD models aligned more with human data than choline-deficient models, particularly in linoleic acid metabolism, unsaturated fatty acid biosynthesis, cholesterol metabolism and PPAR signaling.

The addition of sugar water to HFDs or WDs, with or without CCl_4_, led to profiles more closely aligned with humans regarding glucose metabolism dysregulation (glycolysis or gluconeogenesis) and lipid remodeling (fatty acid elongation). These molecular changes are probably attributable to the sugar water promoting the ChREBP/SREBP1-mediated de novo lipogenesis pathway^7,27,30^. Despite these similarities, very few models (mainly HFD, WD and HFD/WD2%-AMLD and LDLR-CDHFHCD) closely recapitulated (with >90% agreement) the burden of ‘early disease development’ (metabolic) DRP changes observed in human subjects.

**Fig. 5:**
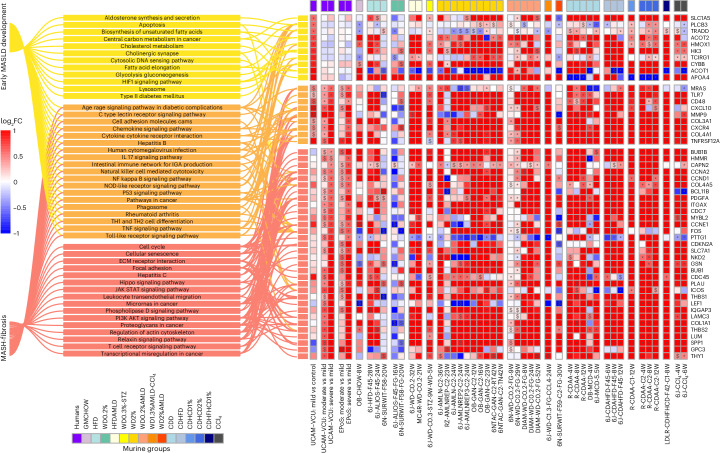
Highly performing genes in human and murine MASLD. Selection of statistically significant DEGs (complete list in Supplementary Table 4) enriching statistically significant modulated pathways (complete list in Fig. 3), thus being highly biologically relevant genes associated with the different stages of MASLD development or progression. To assess the statistical significance between the compared groups, a Wald test statistic (two-sided hypothesis testing) was deployed to compare the coefficients of explanatory variables in a regression model, representing the gene expression differences among the compared groups. The human and murine datasets are represented in a color-scale matrix showing the log2FC. * and $ symbols denote statistical significance ($, *P* < 0.05; *, adjusted *P* < 0.05 (Benjamini–Hochberg correction)). As the graphic legend indicates, all models were grouped according to their macro-categories. Source data

Applying more restrictive filtering to focus on significant DEGs enriched in significant pathways (see Fig. 5), we identified a subset closely linked to various stages of MASLD development and progression. This selected list of biologically relevant genes includes enzymes, cytochromes, transporters, receptors, signal transducers, adaptor proteins and proteins involved in inflammation, extracellular matrix remodeling and cell proliferation or differentiation. It demonstrates that WD, WDAMLD and most choline-deficient dietary models achieve the highest congruence with human MASLD at the transcriptomic level.

These transcriptomics data suggest that most models (apart from GMCHOW, HFD and HFDAMLD, and some choline-deficient models) generally replicate the gene expression dysregulation observed in progressive human MASH. However, human fibro-inflammatory-related transcriptional patterns did not consistently predict murine histological fibrosis, and metabolic pathways did not entirely align with human MASLD. Notably, the analysis of diet macro-clusters and specific diet formulations within the same cluster revealed significant differences in their ‘early disease’ (metabolic) transcriptional responses. This underscores the significance of dietary composition, especially in experiments targeting specific metabolic processes.

### The MHPS is a tool to rank murine models mimicking human MASLD

**Fig. 6:**
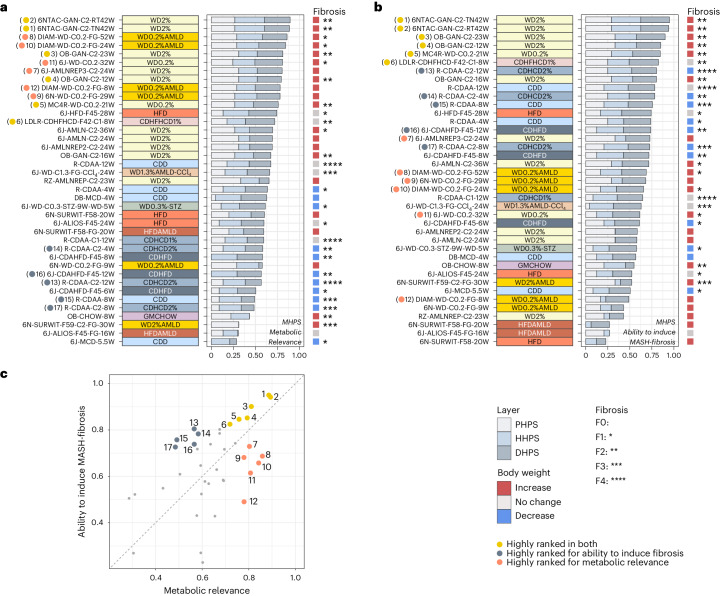
The MHPS—metabolic relevance and progressive MASLD. **a**,**b**, Comparison of the MASLD models, performed based on the MHPS that incorporates the PHPS (details in Supplementary Table 5), the HHPS (details in Supplementary Table 6) and the DHPS (see Supplementary Fig. 4). The average of these normalized scores (MHPS) ranks the murine models (from high to low) based on their metabolic relevance (**a**) or their ability to induce MASH-fibrosis (**b**). A detailed description of the different components is provided in Extended Data Figs. 3 and 4, respectively. For both **a** and **b**, the total length of the horizontal bars indicates the MHPS, while the length of the stacks within each bar indicates the relative contribution from the three evidence layers: PHPS, HHPS and DHPS. A reference panel to the right indicates BW (significant increases in red; decreases in blue) and fibrosis score (*). Macro-categories are indicated by the color panel to the left of the plots. **c**, Correlation among the two MHPS outputs (‘metabolic relevance’ vs ‘ability to induce MASH-fibrosis). Specific models are highlighted based on their performance within the two rankings. Yellow dots represent models that score high with both rankings and represent the best approximation to human MASH. Red dots are models that score highly for metabolic relevance but are less relevant for MASH-fibrosis. Grey dots are models that score highly for MASH-fibrosis but have less metabolic relevance. Panels **a** and **b** provide a specific reference to the position in the scatter. Source data

**Extended Data Fig. 3:**
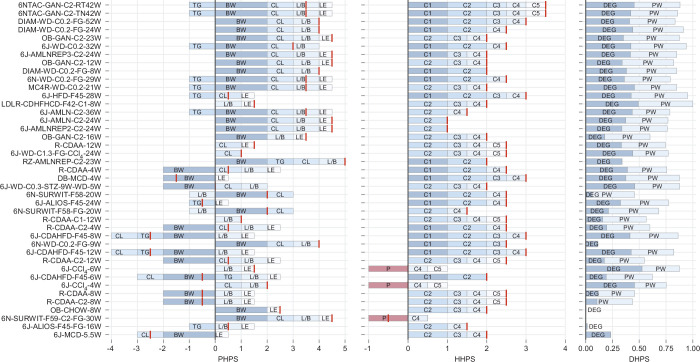
Details of the MHPS components (PHPS, HHPS, DHPS) – Metabolic Relevance. Bar plots show the detailed composition of the MHPS components (PHPS, HHPS, and DHPS) utilised for the output focusing on metabolic relevance. Models are ordered globally according to the MHPS, where each barplot gives an overview of the total PHPS, HHPS or DHPS, the associated features, and their corresponding scores (see Supplementary Tables 5 and 6 and Supplementary Fig. 4 for an overview of the scoring strategies). For each model, the total scores of PHPS and HHPS are represented by red vertical lines to overcome the fact that some features are scored negatively. For PHPS and HHPS, the length of the stacked bars represents the scores’ absolute values (before normalising to the interval 0-1). DHPS directly shows the normalised score. Feature scores included are abbreviated as follows: **PHPS:** BW = Body weight, TG = Triglycerides, CL = Cholesterol, L/B = Liver Weight/Body weight (%), LE = Liver enzyme levels of ALT & AST; **HHPS**: C1 = Topography of histological lesions, C2 = Type of steatosis, C3 = Hepatocyte ballooning, C4 = Lobular inflammation, C5 = Mallory-Denk bodies; **DHPS**: DEG = Differentially Expressed Genes, DRP = Differentially Regulated Pathways. Source data

**Extended Data Fig. 4:**
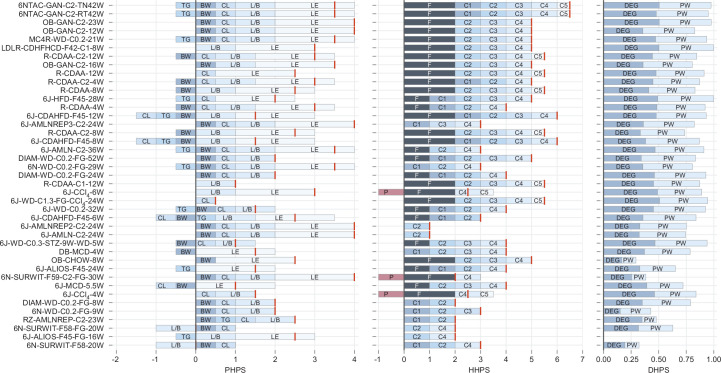
Details of the MHPS components (PHPS, HHPS, DHPS) – Ability to induce MASH-Fibrosis. Bar plots show the detailed composition of the MHPS components (PHPS, HHPS, and DHPS) for the output focusing on the ability to induce MASH-Fibrosis. Models are ordered globally according to the MHPS, where each barplot gives an overview of the total PHPS, HHPS or DHPS, associated features, and corresponding scores (see Supplementary Tables 5 and 6 and Supplementary Fig. 4 for an overview of the scoring strategies). For each model, the total scores of PHPS and HHPS are represented by red vertical lines to overcome the fact that some features are scored negatively. For PHPS and HHPS, the length of the stacked bars represents absolute scores' absolute values (before normalising to the interval 0-1). DHPS shows the normalised score directly. Feature scores included are abbreviated as follows: **PHPS**: BW = Body weight, TG = Triglycerides, CL = Cholesterol, L/B = Liver Weight/Body weight (%), LE = Liver enzyme levels of ALT & AST; **HHPS**: C1 = Topography of histological lesions, C2 = Type of steatosis, C3 = Hepatocyte ballooning, C4 = Lobular inflammation, C5 = Mallory-Denk bodies, F(C6) = Fibrosis; and for DHPS: DEG = Differentially Expressed Genes, DRP = Differentially Regulated Pathways. Source data

The typical subjective method of choosing a murine model for MASLD research is often influenced by standard laboratory practices and constrained by available resources. To provide a more reliable approach for selecting the most suitable models for preclinical research, we developed the MHPS. This data-driven bioinformatics pipeline integrates transcriptomic (drug set enrichment analysis (DSEA) human proximity score, DHPS; referenced in Supplementary Table 4), phenotypic (phenotype human proximity score, PHPS; detailed in Supplementary Table 5) and histopathologic (histopathology human proximity score, HHPS; outlined in Supplementary Table 6) comparative analyses of murine models with human disease. Consequently, the MHPS system ranks murine models based on their congruence with human MASLD in terms of metabolic (illustrated in Fig. 6a; details in Extended Data Figure 3) and/or fibro-inflammatory (shown in Fig. 6b; details in Extended Data Figure 4) profiles.

The MHPS identified the WD0.2%, WD2% and WD0.2%AMLD diets as the most effective in mirroring the metabolic burden of human MASLD (Fig. 6a and Extended Data Figure 3). These diets were highly scored for their role in inducing obesity and dyslipidemia, liver damage (LW:BW% ratio, increased AST and ALT), histological activity (development of steatosis, lobular inflammation and/or ballooning; Fig. 2) and gene expression (Fig. 4 and Supplementary Table 4; under ‘early disease development’ and ‘all disease stages’). The GAN-C2 (ref. ^17^) (ambient and thermoneutral conditions) and the Diamond WD0.2%AMLD models ranked highest. Genetically modified animals (ob/ob, MC4R KO) fed with WDs and the WD1.3%AMLD-CCl_4_ model also scored well, with the additional benefit of a shorter induction period (21–24 weeks) required to develop significant (F2+) fibrosis. Except for CDHFHCD1%-fed LDLR KO mice (8 weeks) and CDAA-fed rats (12 weeks), most choline-deficient dietary models had a lower metabolic ranking, indicating their limited capability to fully replicate the metabolic burden of human MASH.

We then applied the MHPS to evaluate the progression of fibrosing MASH (Fig. 6b and Extended Data Figure 4). This assessment included factors such as liver damage (LW:BW% ratio and increased AST and ALT), histology (MASH with significant fibrosis; Fig. 2) and gene expression patterns outlined in Fig. 4 and Supplementary Table 4, specifically focusing on ‘all disease stages’ and ‘disease progression’. The MHPS highly ranked models like the GAN-C2 in C57BL6/NTac and ob/ob mice, MC4R KO mice on a WD0.2% diet, CDHFHCD1%-fed LDLR KO mice (over 8 weeks) and CDHCD2% fed rats (over 12 weeks); in terms of liver histopathology outcomes, each of these models developed significant (F2–F4) fibrosis. The only diet inducing F4 fibrosis within a 12-week period was CDAA (with or without 1–2% cholesterol) in rats. Despite its high performance in individual MHPS components such as HHPS (developing F3 fibrosis) and DHPS, WD1.3%AMLD-CCl_4_ (ref. ^21^) was penalized in the overall MHPS because of variability in mouse phenotypes reflected in the PHPS. Most other HFD, HFDAMLD, WD and WDAMLD models scored lower in this ranking, partly due to limited fibrosis. Notably, most choline-deficient models (CDD, CDHCD, CDHFD) did not rank highly despite inducing significant (F2–F4) fibrosis when considering all MHPS components together.

**Extended Data Fig. 5:**
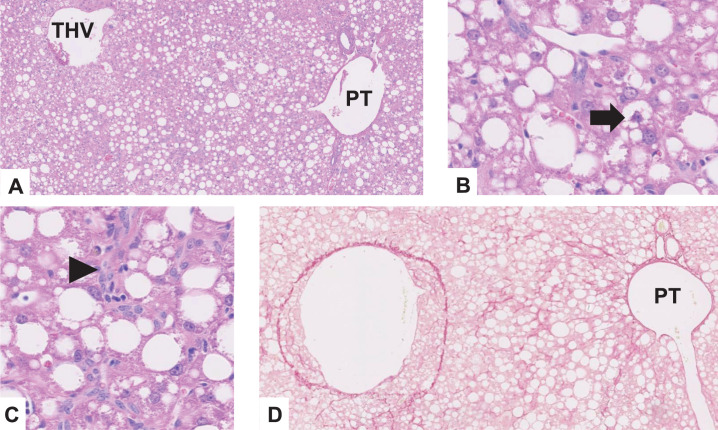
Representative images of MASLD histopathology features in the 6NTAC-GAN-C2-TN42W model. **a**. Panlobular distribution of steatosis, grade 3, H&E stain, x50; **b**. Ballooned hepatocyte (arrow), H&E stain, x200; **c**. Necroinflammatory focus (arrowhead), H&E stain, x200; **d**. Periportal and extensive sinusoidal fibrosis, stage 2, Sirius red stain, x50 (THV terminal hepatic venule, PT portal tract).

**Extended Data Fig. 6:**
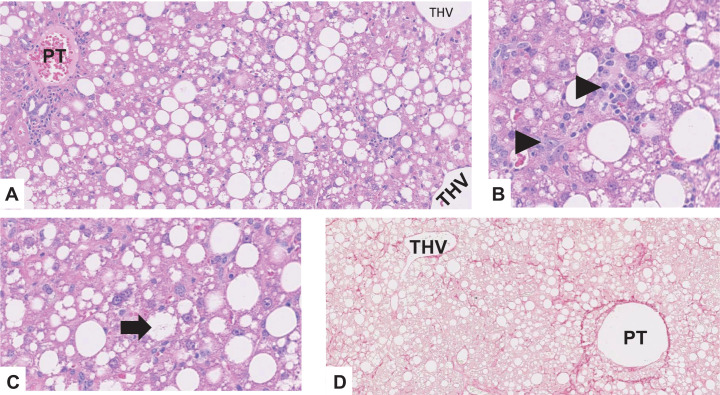
Representative images of MASLD histopathology features in the 6NTAC-GAN-C2-RT42W model. **a**. Panlobular distribution of steatosis, grade 3, H&E stain, x50; **b**. Necroinflammatory foci (arrowheads), H&E stain, x200; **c**. Ballooned Hepatocyte (arrow), H&E stain, x200; **d**. Periportal and perisinusoidal fibrosis, stage 2, Sirius red stain, x50 (THV terminal hepatic venule, PT portal tract).

**Extended Data Fig. 7:**
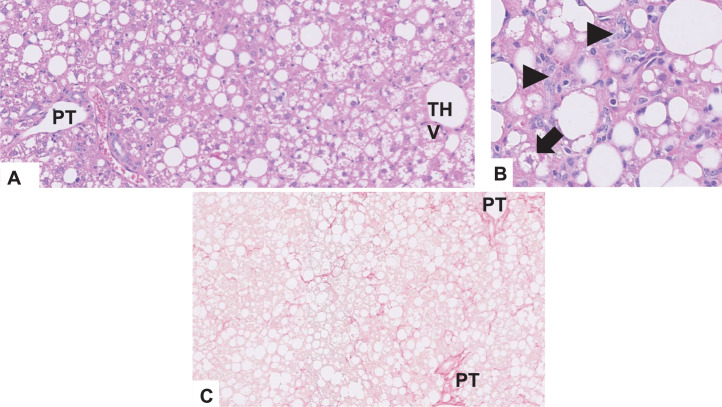
Representative images of MASLD histopathology features in the AZ-OB-GAN-23W model. **a**. Panlobular distribution of steatosis, grade 3, H&E stain, x50; **b**. Non-classical ballooned hepatocyte (arrow) and necroinflammatory foci (arrowheads), H&E stain, x200; **c**. Periportal and perisinusoidal fibrosis, stage 2, Sirius red stain, x50 (THV terminal hepatic venule, PT portal tract).

**Extended Data Fig. 8:**
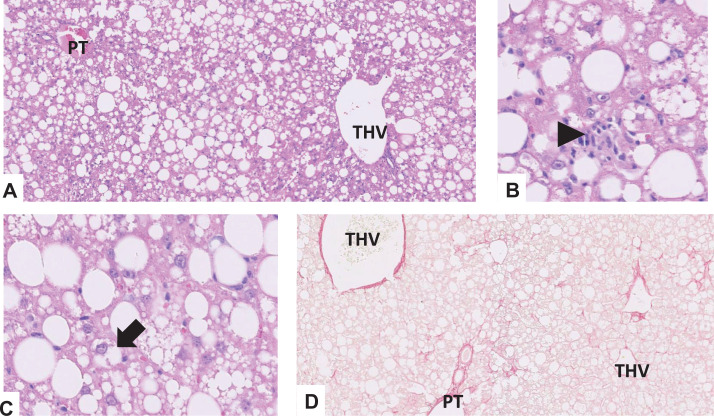
Representative images of MASLD histopathology features in the OB-GAN-C2-12W model. **a**. Panlobular distribution of steatosis, grade 3, H&E stain, x50; **b**. Necroinflammatory focus (arrowhead), H&E stain, x200; **c**. Ballooned hepatocyte (arrow), H&E stain, x200; **d**. Periportal and perisinusoidal fibrosis, stage 2, Sirius red stain, x50 (THV terminal hepatic venule, PT portal tract).

**Extended Data Fig. 9:**
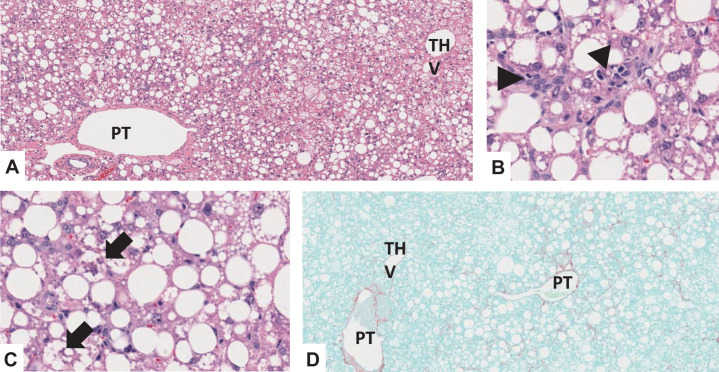
Representative images of MASLD histopathology features in the MC4R-WD-C0.2-21W model. **a**. Panlobular distribution of steatosis, grade 3, H&E stain, x50; **b**. Necroinflammatory foci (arrowheads), H&E stain, x200; **c**. Ballooned hepatocytes (arrows), H&E stain, x200; **d**. Periportal and perisinusoidal fibrosis, stage 2, Sirius red fast green stain, x50 (THV terminal hepatic venule, PT portal tract).

**Extended Data Fig. 10:**
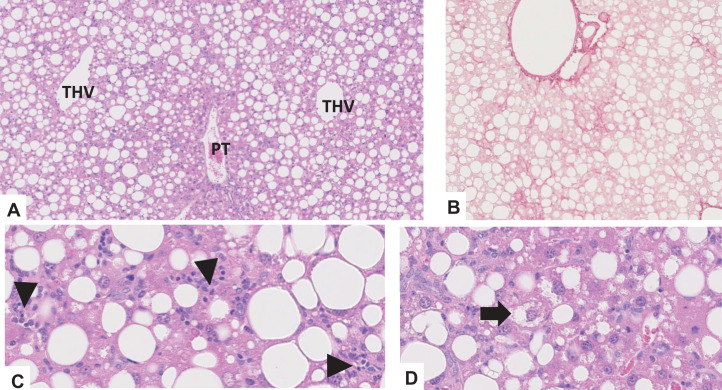
Representative images of MASLD histopathology features in the LDLR-CDHFHCD-F42-C1-8W model. **a**. Panlobular distribution of steatosis, grade 3, H&E stain, x50; **b**. Periportal and perisinusoidal fibrosis, stage 2, Sirius red stain, x50; **c**) Necroinflammatory foci (arrowheads), H&E stain, x200; **d**. Ballooned hepatocyte (arrow), H&E stain, x200 THV terminal hepatic venule, PT portal tract).

Subsequently, we compared the two sets of rankings to identify study designs that most effectively model the complete spectrum of MASLD features, as shown in Fig. 6c. Our comparison confirmed that WDs predominantly replicated the metabolic aspects of the disease, while choline-deficient diets better featured the pro-fibrotic components of MASH. Interestingly, several specific murine models scored highly in both rankings, making them the best choices for replicating both metabolic and fibro-inflammatory characteristics of human MASLD. These standout models included C57BL6/NTac or ob/ob mice on a GAN-C2 diet, MC4R KO mice on a WD0.2% diet and LDLR KO mice undergoing a CDHFHCD1% diet challenge. Representative histopathology images of these highly ranked models are provided in Extended Data Figs. 5–10.

In conclusion, although no single model perfectly matched human MASLD, our analysis identified several study designs that closely approximate various aspects of the disease burden.

### Key experimental components contributing to MHPS ‘metabolic relevance’ and ‘ability to induce MASH-fibrosis’ outputs

To gain a deeper understanding of the specific parameters of the study designs (outlined in Supplementary Table 1) that influenced the rankings of the murine models, we used a partial least squares regression (PLSR) analysis. This analysis explored the connection between the study designs and the MHPS outputs ‘metabolic relevance’ and ‘ability to induce MASH-fibrosis’. Our focus was on mouse models fed choline-sufficient diets, as the available data for rat models and choline-deficient dietary models lacked the necessary detail and granularity required for a robust PLSR analysis.

**Fig. 7:**
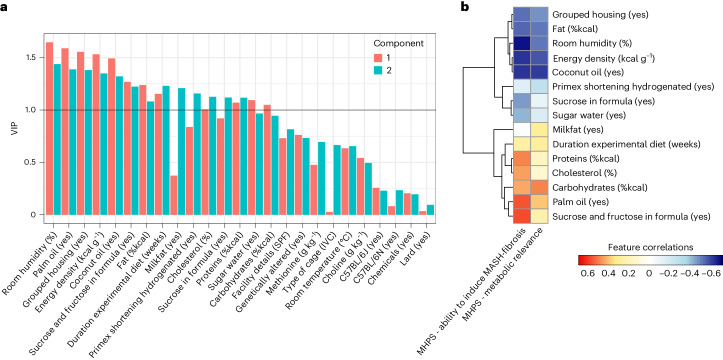
Key experimental components contributing to MHPS ‘metabolic relevance’ and ‘ability to induce MASH-fibrosis’ outputs. Relationship between study design parameters and the MHPS of metabolic relevance and ability to induce MASH-fibrosis evaluated by PLSR. **a**, VIP for each study parameter (among those detailed in Supplementary Table 1) for the two components of the model. Parameters contributing the most to the PLSR model are characterized by having VIPs > 1 (black solid line) in any of the two components of the model. **b**, Clustered heatmap of the most influential study parameters (VIP > 1), indicating their correlation with the MHPS metabolic relevance and ability to induce MASH-fibrosis (color-scale matrix showing a positive correlation represented in red; negative correlation represented in blue). Source data

The variable importance in projection (VIP) score was used to identify the most influential parameters in the PLSR model. We set a VIP threshold of at least 1 in one component (Fig. 7a) to determine these key parameters. These were then further examined for their correlation with the MHPS outputs ‘metabolic relevance’ and ‘ability to induce MASH-fibrosis’ to understand their impact on study outcomes, depicted in Fig. 7b. The analysis revealed that some study outcomes negatively correlated with certain housing conditions, such as room humidity and grouped housing. Additionally, aspects of the dietary formula like energy density, percentage of fat and the use of coconut oil as a fat source also showed negative correlations. Conversely, the duration of the dietary challenge and specific nutritional formula features were positively correlated with MHPS. Noteworthy among these positively correlated variables were the relative nutrient composition (%kcal) in terms of proteins, cholesterol, carbohydrates, the use of palm oil as a fat source, and the combination of sucrose and fructose as carbohydrate source. Interestingly, the use of sucrose alone and sugar water (in the AMLD approaches) exhibited a negative correlation with the MHPS output for the ‘ability to induce MASH-fibrosis’.

These results highlight critical features of the study designs that drive disease outcomes and provide insights into how additional model optimization could occur.

### WDs allow the study of therapeutic interventions for BW loss and MASH amelioration

**Fig. 8:**
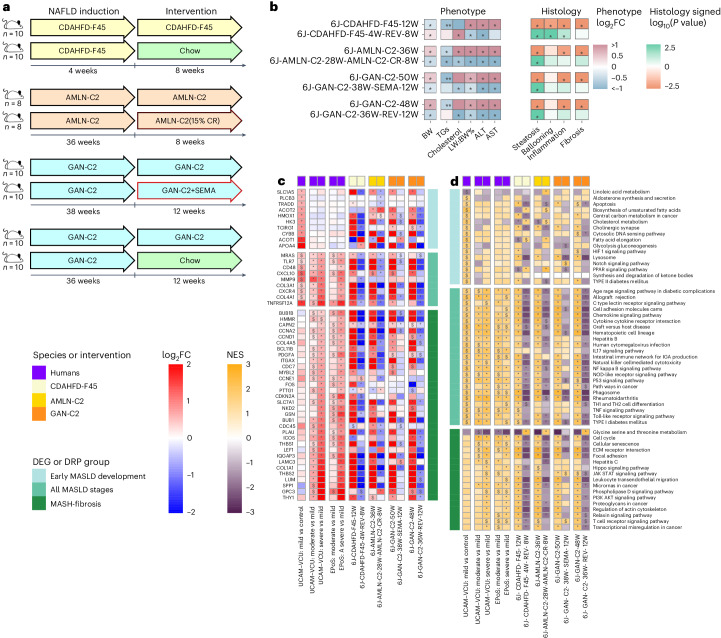
Effects of treatments in a selection of WD and choline-deficient models. Response of the best-ranked diet (GAN-C2), another WD (AMLN-C2) and a CDHFD (CDAHFD-F45) to treatments mimicking lifestyle intervention (CR, caloric restriction; REV, chow reversal) and semaglutide pharmacological treatment (SEMA, 30 nmol per kg per day). **a**, Simplified study designs (details in Supplementary Table 7). **b**, Effect on the phenotype and histology. Phenotypic changes observed in the treatment or dietary models compared to their matched dietary models or controls, respectively, were profiled as log_2_FC across measures of BW, blood TGs and cholesterol, LW:BW%, and ALT and AST. The red–blue color gradient indicates the level of increase–decrease of the measure in the models compared to their respective controls; * indicates statistical significance (*P* < 0.05; two-sided Mann–Whitney *U*-test). For the histological changes, the Mann–Whitney *U*-test was used to calculate *P* values for the differences in the ordinal scores (*P* < 0.05 are shown with *). The color scale indicates the signed *P* value: −log_10_(*P* value) for up-regulation and +log_10_(*P* value) for down-regulation. **c**, Effect of treatments on a selection of biologically relevant DEGs as described in Fig. 5. The human and murine datasets are represented in a color-scale matrix showing the log_2_FC. * and $ symbols denote statistical significance (two-sided Wald test statistic and adjustment for multiple testing using the Benjamini–Hochberg correction; $: *P* < 0.05; *: adjusted *P* < 0.05). **d**, Effect of treatments on pathways as described in Fig. 3. FGSEA calculated a NES for each pathway and determined statistical significance using permutation testing (two-sided), adjusting for multiple comparisons to control the FDR. The human and murine datasets are represented in a color-scale matrix showing the NES. $, *P* < 0.05; *, FDR < 0.05). Source data

To gain further insights into the significance of the models that the MHPS ranked highly, we used the same phenotyping approach to assess their responsiveness to dietary and therapeutic interventions to promote weight loss. These interventions included chow reversal (12 weeks of chow reversal following 38 weeks on the GAN-C2 diet, or 8 weeks of chow reversal after 4 weeks on the CDHFD45% diet), calorie restriction (8 weeks of calorie restriction following 28 weeks of AMLN-C2 diet) and treatment with the GLP-1 receptor agonist semaglutide (12 weeks of semaglutide following 38 weeks of the GAN-C2 diet)^31–34^. Additional information regarding these study designs is provided in Supplementary Table 7 and illustrated in Fig. 8a.

Figure 8 and Supplementary Tables 7 and 8 demonstrate that all interventions (chow reversal, calorie restriction, semaglutide) substantially improved liver damage (evidenced by LW:BW% ratio, transaminases, steatosis) and reversed many effects of the MASLD-inducing diets on the DEGs and DRPs modulated in human MASH (as described in the sections above). However, it is essential to note that significant improvements in hepatocyte ballooning and inflammation were not observed in mice on AMLN-C2 and GAN-C2 diets. This suggests that longer treatment durations may be necessary to achieve substantial therapeutic effects. Interestingly, none of the treatments resulted in a significant improvement in fibrosis. Weight and dyslipidemia improved only in MASLD induced by AMLN-C2 and GAN-C2 diets. Notably, the GAN-C2 model effectively mimicked the lack of fibrosis improvement (despite an improved activity score) recently reported for semaglutide in human trials^35^. By contrast, chow reversal following the CDHFD diet paradoxically increased BW and cholesterol levels. This finding highlights the superiority of WD2% models like GAN-C2 for treatments targeting BW and/or metabolic pathways.

## Discussion

Generating a murine model that fully recapitulates MASLD pathophysiology and mimics human disease is the holy grail of preclinical research for which there are too many candidates and no consensus^16^. Although various preclinical models have been suggested to replicate human MASLD, a thorough comparative study that aligns these animal models with human clinical outcomes and assesses their closeness to human disease pathophysiology through ‘omics’ approaches is still missing. Therefore, we performed a 3Rs (replacement, reduction and refinement) -compliant retrospective review of commonly used murine MASLD models available to the LITMUS Consortium and collaborators. These models were evaluated and ranked based on their alignment with three critical features of MASLD in humans: clinical metabolic phenotype, liver histopathology and liver transcriptome benchmarked against human transcriptomic changes.

The evaluation criteria for these models were established in advance and quantified using human proximity scores tailored to assess phenotype (PHPS), histopathology (HHPS) and transcriptome (DHPS). These distinct scores were subsequently integrated to create the MHPS. This innovative and insightful tool ranks murine models in terms of their resemblance to human cohorts with biopsy-confirmed NAFLD/MASLD^25,26^.

Our study provides several important outcomes and unique insights.

A key strength of our study lies in the uniformity of methodology applied across all models for staining and scoring liver histopathology. Additionally, we have developed an original approach for interpreting murine histology: our proposed HHPS is grounded in the canonical parameters (such as steatosis, hepatocyte ballooning, lobular inflammation and fibrosis) used by established human NAFLD/MASLD scoring systems (CRN and SAF)^25,26^. However, the HHPS introduces a unique qualitative scoring approach, focusing on how the morphological features of liver tissue in mice mirror the pathology observed in humans, as detailed in Supplementary Table 6. This approach is designed to complement, but not replace, the canonical scores, which are more typically used to assess the impact of specific treatments on histology. Our study also details the development of hepatocyte ballooning in murine models, addressing previous debates and uncertainties surrounding this histological feature^16^.

Our extensive comparison and ranking of preclinical models identified a short list of those that most closely align with human disease progression; however, none are able to fully replicate all aspects of human MASLD. This finding may reflect the inherent complexity and diversity of human disease and the fundamental physiological differences between humans and murine models; these differences encompass lipid metabolism, energy expenditure, circadian rhythms and eating patterns. Furthermore, how animals react to various diets, manifesting specific metabolic traits, exhibiting incomplete histological damage (for instance, hepatocyte ballooning) or responding to medications (including unique reactions or BW changes that do not mirror human responses), can influence the direct comparability between murine models and human disease^12–14,35–38^.

We have characterized molecular transcriptomic changes in each murine model, introducing DHPS to evaluate their resemblance to human NAFLD/MASLD transcriptomic alterations. This molecular characterization is crucial for selecting the most appropriate preclinical models for specific mechanistic or pharmacological studies, such as targeting a particular gene or pathway. Moreover, our findings highlight that relying solely on transcriptomics does not accurately predict disease progression or fibrosis. Although transcript changes in HFD and most WD and AMLD models align with differentially modulated pathways and genes in MASH progression, like those markers indicating stellate cell activation or collagen deposition, they might not correspond with significant histologic fibrosis. This discrepancy is vital to consider in disease progression studies and therapeutic interventions whereby conclusions are based only on gene expression changes. For instance, the pharmacological treatment with semaglutide in the GAN-C2 model did not improve fibrosis histologically despite enhancing these pathways at the transcriptomic level, as seen in human trials^39^.

Most importantly, our research has identified a particularly valuable subset of murine models accurately reflecting human MASLD in terms of metabolic and fibro-inflammatory aspects. We found that dietary formulas rich in fat, refined carbohydrates (notably the combination of glucose and fructose) and cholesterol effectively replicate many facets of human MASLD, including fibrosis. A key observation is that diets with high cholesterol levels (up to 2%) accelerate disease progression and guarantee significant fibrosis.

Upon examining diet compositions more closely, WD and WDAMLD variations, with or without a low dose of CCl_4_, emerge as the most well-rounded options for most preclinical studies. These diets induce metabolic disturbances and lead to progressive MASH, mirroring the progression in human MASLD. Significant fibrosis (F2 or higher) can be induced by combining a high cholesterol content (2%) with extended duration (over 40 weeks) and/or genetically altered (obese) backgrounds. In our evaluation of various study designs, the most effective performance was observed with the GAN-C2 diet fed to C57BL6/NTac or ob/ob mice. Regarding clinical translatability, the GAN-C2 model responded well to therapeutic interventions, showing improvements in metabolism, liver damage and gene expression. By contrast, choline-deficient diets (like CDD, CDHFD and CDHCD) achieved F2+ fibrosis in shorter time frames (12 weeks or less) but lacked a significant metabolic phenotype. Compared to the WD, these diets did not offer a substantial advantage in closely replicating human MASH-fibrosis regarding histopathology and gene expression, as illustrated in Fig. 6b and Extended Data Figure 4. However, they may benefit specific research objectives, such as testing anti-fibrotic treatments within reasonable time frames. The performance of the WD1.3%AMLD-CCl_4_ (ref. ^21^) diet lies somewhere between WDs and choline-deficient dietary models. The translational potential of choline-deficient dietary models can be partly enhanced by using rat models and/or altering cholesterol metabolism. In our study, the choline-deficient dietary model with relatively better metabolic relevance was CDHFHCD1% given to LDLR KO mice for 8 weeks.

Our research also indicates the potential for further optimization of murine models. Although most model clusters effectively modulate key pathways integral to MASH progression, their alignment with human metabolic pathways is only partial. Building on the models we have identified as closely mirroring human metabolic characteristics and disease progression, a unique opportunity exists to further refine study designs to enhance the models’ relevance. We observed that even minor variations in dietary formulas significantly impacted the regulation of metabolic pathways. PLSR and correlational analyses with the MHPS (Fig. 7) suggest that a combination of high cholesterol content, palm oil as a fat source and a carbohydrate mix of fructose plus glucose could be the basis for further dietary formula optimization. This approach could lead to more accurate models replicating the complex metabolic interactions seen in human MASLD.

Adjustments can be considered with regards to (1) modifying the energy density of MASH-inducing diets: incorporating sugar water (the AMLD approach) and the high-energy density of diets were found to correlate negatively with fibrosis endpoints. This suggests that rodents’ mechanisms of adapting to nutrient intake^40^ might delay disease progression. (2) Improving nutrient composition of MASLD-inducing diets: the study outcomes correlated with the relative nutrient composition suggest that a reduction in fat content (to or below 40%) and a more balanced inclusion of proteins and refined sugars could enhance both metabolic outcomes and fibrosis, potentially leading to a more physiological modulation of lipid and cholesterol pathways in agreement to human MASLD. (3) Considering housing parameters: grouped housing and room humidity also negatively correlated with MHPS. Thermoneutrality reduces energy expenditure, impairs systemic metabolism and might promote MASLD in chow-fed animals while accelerating the development of liver damage with a HFD^41–45^. By contrast, the phenotype of GAN-C2, which induces a more severe MASH phenotype than HFD at ambient temperature, did not differ significantly when evaluated at thermoneutrality (Figs. 2, 3 and 6), questioning the practicality of its routine use due to organizational complexity^16,41–45^. (4) Duration of diet, but not the use of genetically modified animals, positively correlated with disease outcomes. Genetically modified models of obesity (for example, ob/ob, MC4R KO mice, ZSF1 rats) and dyslipidemia (for example, LDLR KO mice) accelerated liver histopathology damage and intensified some metabolic features. Despite the convenience of these genetic backgrounds for certain studies (for example, to study MASLD association with cardiovascular disease or obesity) and as quick models of MASH progression, caution is recommended, given that they do not adequately recapitulate human disease (given the low frequency of such mutations in human MASLD), they can feature an impairment in key pathophysiological pathways occurring in wild-type animals (for example, in Extended Data Figure 1, the transcriptomic profiles of ob/ob and db/db mice are different from those of wild-type animals) and their genetic perturbations may influence the response of the mouse to specific treatments (including confounding ‘off-target’ effects). However, genetically altered animal models can help to study the impact of genetic obesity or to explore the association of MASLD with atherosclerosis. (5) The addition of chemicals (for example, STZ or low-dose CCl_4_) shortens the duration of the study and improves specific disease outcomes (like diabetes or fibrosis) but does not significantly enhance the overall relevance of the study designs to human conditions.

Other variables not evaluated in the present study that could provide an opportunity to improve model performance include investigating the impact of gender and/or microbiota on study outcomes. Additionally, exploring the effectiveness of these designs in other species, such as rats or larger mammals, could provide valuable insights. There is also scope for testing these models in genetic backgrounds more prone to obesity and liver damage. The use of humanized mice is another approach worth exploring; however, it is essential to acknowledge that high costs and technical and/or biological limitations may restrict their widespread use in the immediate future^11,46–48^. These future refinements could be crucial in enhancing the applicability and relevance of currently studied murine models in MASLD research.

Finally, our study significantly contributes to the field by proposing a standard for consensus in selecting murine models and offering an extensive dataset to facilitate future research. To address the challenges of standardization and consistency^16^ and to prevent the proliferation of new models, we recommend that any claims regarding the superiority of proposed preclinical models over established ‘gold standards’ should be rigorously tested. This approach is essential to maintain clarity and avoid further confusion in the scientific literature.

This study, while comprehensive, does face certain limitations. (1) The retrospective design constrained our capabilities, for instance, in harmonizing study designs regarding fasting duration, sample sizes and statistical power. (2) Most of the studies were conducted on male rodents, preventing us from exploring gender differences in hepatic metabolism and MASLD, as well as the impact of diets in female animals; this highlights the need for work in female rodents as an important area for future research. (3) Available funds and resources limited our model selection: expanding our screening to include the vast array of study designs documented in the literature and female animals was technically and financially impractical^15^. (4) We could not examine the relevance of the murine models included to heterogeneous subtypes of human MASLD, such as pediatric or lean MASLD, or those involving genetic risk variants impacting phospholipid metabolism or lipoprotein secretion. The murine models tested also did not extend to advanced disease stages like cirrhosis, portal hypertension or hepatocellular carcinoma. (5) Of the various compounds currently undergoing human trials, only data and samples from mice treated with semaglutide were available for our study. (6) Insufficient information on glucose metabolism in most models precluded a systematic investigation of insulin resistance. (7) The impact of thermoneutrality has been explored only in the GAN-C2 model; therefore, its impact on disease outcomes cannot be generalized to all dietary models.

Despite these limitations, the thoroughness of our study and the systematic approach used in ranking the murine models render it a unique and valuable resource for the MASLD research community. This work lays the groundwork for future research to enhance preclinical murine models and achieve a consensus in the field.

In summary, our study reveals the extent to which the phenotype, liver histology and liver transcriptome of commonly used murine models of MASLD resemble human disease. Moreover, this study provides a comprehensive resource to explore how murine models recapitulate essential features of MASLD pathophysiology (for example, metabolic pathways, stellate cell activation, fibrosis development and so on), enabling optimal model selection based on the specific needs of a study. The MHPS rankings show that WD models emerge as the most balanced in terms of metabolic, histologic and transcriptomic similarities to human disease. However, only a few models exhibit MASH with significant fibrosis (F2 or higher), which typically necessitates extended durations, high cholesterol content and/or the use of genetically altered backgrounds.

In conclusion, our research provides a crucial foundation for the field, delineating the strengths and weaknesses of various prevalent MASLD models. This knowledge enables researchers to make informed decisions when choosing an experimental model that best aligns with human disease state and the specific requirements of their projects. Future studies should build on this work to refine MASLD murine models, enhancing their translational relevance and applicability to the diverse pathophysiology observed in human MASH.

## Methods

### Animal experiments

#### Training cohort

In this retrospective study, we compiled data and liver formalin-fixed paraffin-embedded tissues from 509 mice and 89 rats (39 MASLD models and CCl_4_ as positive control for isolated fibrosis). These experimental designs represent a combination of diet, species, genetic background, wild-type or genetically altered animals, time points, sex and housing room temperature compared to their own control diets. A detailed description of each study protocol is described in Supplementary Table 1. Only models with centralized histology assessment (detailed below), transcriptomics data (detailed below) and phenotypic information were included in the analyses. Standard metabolic biochemistry (serum triglycerides, cholesterol, ALT, AST) and liver function tests were performed. The data included anthropometric measures (BW; LW; LW:BW%), breeder, strain, and housing and feeding design (for example, room temperature, type of facility and cages, health status of the animal facility).

For the 6J-WD-C1.3-FG-CCl_4_-24W model, known as the FAT (fibrosis and tumors)-MASH rodent model (established in the Friedman laboratory^21^), 6-week-old male C57BL/6J mice were purchased from Jackson Laboratories. Five mice per cage were housed in a *Helicobacter*-free room in a 12 h light, 12 h dark cycle and weighed once weekly. CCl_4_ was purchased from Sigma-Aldrich. CCl_4_ was freshly dissolved in corn oil at a final concentration of 5% before injection. The final dose of pure CCl_4_ was 0.2 μl g^−1^ of BW, delivered intraperitoneally once per week, starting from initiation of the WDAMLD feeding, and continued for 24 weeks.

For the 6J-WD-C0.3-STZ-9W-WD-5W model, male C57BL/6J mice were purchased from Shanghai Lingchang Laboratory Animal Co. LTD. Two days after birth, these mice were administered a single dose (subcutaneous injection) of 200 µg STZ (Sigma, St. Louis, MO, USA). Upon reaching the age of 4 weeks, the mice were subjected to WD for 5 weeks. Details in Supplementary Table 1.

As a positive control for fibrosis, we used experiments in which peri-central fibrosis was induced by repeated CCl_4_ injury. Mice were given CCl_4_ (Sigma) dissolved in olive oil (Sigma) intraperitoneally at a dose of 0.75 ml kg^−1^ of BW three times a week for 6 weeks. The age-matched control group received olive oil as a vehicle. At the end of the study, mice were humanely killed with carbon dioxide and liver tissue was fixed in 10% buffered formalin for histopathology analysis and snap-frozen in liquid nitrogen for RNA analysis.

### Treatments and intervention cohorts

The ‘Treatments’ cohort consisted of a total of 108 male mice challenged either with AMLN-C2 (36 weeks) or GAN-C2 (36 or 38 weeks) or CDAHFD-F45 (4 weeks) diets and followed by treatment, meaning dietary intervention (that is, calorie restriction (36 weeks) or chow reversal (8 or 12 weeks)) or pharmacological treatment (semaglutide (12 weeks)).

The data quality was comparable to the training cohort; a detailed description of each study protocol is provided in Supplementary Table 7.

### Ethical approvals for all animal experiments

Relevant animal welfare authorities approved all the animal experiments that complied with national and international guidelines (details in Supplementary Tables 1 and 7).

### Human next-generation sequencing datasets

The human cohorts were established according to the recruiting criteria of the old definition (NAFLD/NASH).

The UCAM–VCU super-cohort consisted of two publicly available (E-MTAB-9815, GSE130970) datasets (total of 136 patients) previously described by our teams^27,29^. All patients had a clinical diagnosis of NAFLD and histology scores according to the CRN scoring system^25^. Patients were divided into control (*n* = 4) and NAFLD (*n* = 132) subclustered against fibrosis (mild (F0), *n* = 52; moderate (F1–F2), *n* = 50; severe (F3–F4), *n* = 30).

The EPoS database orginates from a large cohort of NAFLD patients recruited in different European Union institutions, with publicly available next-generation sequencing data (GSE135251); this cohort was previously described by our team^28^. All patients had a clinical diagnosis of NAFLD/MASLD; histology was centrally scored according to the CRN scoring system as previously described^25^. A total of 38 patients from the initial cohort were removed, as they overlapped with the UCAM–VCU dataset. The remaining 168 patients with NAFLD were subclustered against fibrosis (mild (F0), *n* = 47; moderate (F1–F2), *n* = 64; severe (F3–F4), *n* = 57).

A description of the two cohorts is shown in Supplementary Table 3.

The relevant ethics committees (UCAM–VCU Cohort, East of England Research Ethics Committee and Virginia Commonwealth University; EPoS Cohort, multiple ethical committees in the participating countries) approved these studies as detailed in the original publications^27–29^. All patients gave informed consent to use biochemistry, clinical history and samples for research purposes. The principles of the Declaration of Helsinki were followed.

### Murine histology

All murine liver samples were centrally stained and scanned by the Integrated Biobank of Luxembourg (IBBL). Each LITMUS partner shipped unstained formalin-fixed paraffin-embedded tissue slides. The slides were incubated for 30 min at 65 °C and then processed using a Varistain Gemini Slide Stainer. Following deparaffinization in xylene (×2, 5 min each) and rehydration in decreasing alcohols (100%, 95%, 70%; 2 min each) and tap water (1 min), one slide per case was stained as follows. H&E staining: immersion in hematoxylin (4 min), water wash (1 min), 1% acid alcohol (15 s), tap water wash (1 min), bluing reagent for counterstaining (1 min), tap water wash (1 min), 95% alcohol (1 min), alcoholic eosin (20 s), three alcohols (100%; 2 min, 2 min and 3 min, respectively), clearing in xylene substitute (two sets of 1 min and 2 min, respectively) and mounting. Hematoxylin 560 staining solution (Leica, 3801570 each four per case or 3801570 each); alcoholic eosin Y 515 (Leica, 3801616 each four per case or 3801615 each), bluing reagent (Thermo Scientific, Epredia, 6769001), hydrochloric acid 37% (Sigma-Aldrich, 320331). Sirius Red staining: the slides were immersed in picrosirius red solution (VWR K640745, 500 ml) for 30 min, then rinsed in tap water, immersed in three alcohols (100%; 2 min, 2 min and 3 min, respectively), cleared in xylene substitute and mounted.

All stained slides were scanned by IBBL using the Nanozoomer 2.0 HT Slide Scanner; digital images were made available to the LITMUS Histopathology Group in a blinded manner using the CaloPix digital slide platform (TRIBVN Healthcare) for central histological scoring. LITMUS Histopathology Group members were all expert liver pathologists and assessed one H&E-stained and one Sirius Red-stained digital slide per case using the CRN and SAF scoring systems, as previously described^25,26^. Liver pathologists were harmonized to score human MASLD/MASH biopsies in LITMUS; their κ-score regarding interobserver agreement for hepatocyte ballooning has been shown to be substantial (κ = 0.8)^6^. Before scoring the animal model slides, a further round of harmonization took place. All liver pathologists had prior experience in histologically scoring animal MASLD model samples. A single pathologist scored each model.

The LITMUS Histopathology Group also developed an ad hoc scoring system (the HHPS; see Supplementary Table 6) that complements canonical scoring systems, providing a metric of how much the histological lesions in the murine models approximate human MASLD pathology.

### Murine transcriptomics datasets

The murine models/datasets were derived from different LITMUS partners and facilities, and/or collaborators. As summarized in Supplementary Table 1, all models had RNA sequencing (RNA-seq) data, apart from two models profiled using Affymetrix microarray technology (6N-WD-C0.2-FG-9W, 6N-WD-C0.2-FG-29W). For RNA extraction and sample processing, different protocols and kits were used (STAT-60, RNeasy, Trizol), while the sequencing was performed mainly using Illumina’s platforms (HiSeq3000/4000, NextSeq500/550, NOVASeq6000), using single- or paired-end reads (details in Supplementary Tables 1 and 7). The diversity of our dataset is explained by the retrospective design of the experiment. The experimental units have provided different models with samples obtained at different time points, leading to a merged non-standardized dataset regarding libraries, sequence methodologies and protocols.

### Processing RNA-seq and microarray data

The models with microarray data were analyzed with R packages oligo (v.1.46)^49^ and limma (v.3.38.2)^50^, using RMA normalization and PCA analysis evaluating technical metadata. Technical outliers with only one or two samples processed for a given scan date were removed, retaining 13–15 samples for each time point and diet. Differential expression analysis was performed between the control and experimental diets, using linear model fit for each time point (Supplementary Fig. 2).

For all RNA-seq data, FastQC (v.0.11.9; https://github.com/s-andrews/FastQC) was used to test fastq files, and HISAT2 (v.2.1.0) mapped the reads to the human GRCh38, mouse GRCm38 or rat Rnor_6.0 genomes, using default parameters^51^. To proceed to subsequent analysis steps, all samples passed the following criteria: GC% content was approximately 50%, more than ten million reads passed the quality filtering and more than 80% of the reads per sample were mapped to the reference genome. HTSeq^52^ (v.0.11.1) was used for gene counting and the R package biomaRt (v.2.54.0) mapped the ensemble gene IDs to HGNC symbols (Supplementary Fig. 2).

Given that the rodent datasets were produced by different facilities, in different batches and using different sequencing methodologies, we had to apply a batch-effect correction strategy to merge all datasets. First, quantile normalization was applied and then the Bioconductor function COMBAT^53^ from the R package sva (v.3.38.0) removed the batch effect caused by data derived from different units. For the human datasets, batch effects were addressed inside DESeq2 (ref. ^54^) using batch (dataset and gender) as a vector in the design formula (design = ~batch + condition). Differential gene expression analysis was performed with DESeq2 (ref. ^54^) (v.1.26.0) in the rodent models (treatment vs control) and the human comparison groups (mild vs control, moderate vs mild, severe vs mild).

Finally, we selected a standard set of genes expressed in all the models for input in the enrichment analysis of the different rodent models. Applying log_2_(transformed copies per million) (log_2_CPM) normalization (cpm from the R package edgeR v.3.32.1), the abundance of each transcript was normalized against the total number of reads in a sample, leading to high reproducibility of the average expression of housekeeping genes among the different batches. The selection of the standard set of genes expressed in the murine liver was based on the median CPMs derived from the livers of 50 healthy mice downloaded from Expression Atlas (https://www.ebi.ac.uk/gxa/download) and the position where the bimodal distribution for the high and low expressed genes cross (Supplementary Fig. 2; *x*_cross_ = −2). All the genes with Log_2_CPM expression less than −2 in more than 10% of the models were excluded to ensure no substantial differences in the enrichment in the different models.

### Statistical analysis and reproducibility

Each murine experiment was repeated once. Before calculating the statistical significance in R, log_2_ transformation was applied to the phenotypic data. The raw *P* values (each group vs its own control) were calculated for both phenotypic and histological data using the Mann–Whitney *U*-test (wilcox.test, R stats package v.3.6). The Benjamini–Hochberg method was applied to the results of the differential expression analysis to adjust the raw *P* values controlling the false discovery rate^55^. The batch-effect-corrected normalized counts corresponding to each animal were used to produce the principal components (PCs) in the mice and rats PCA plots, respectively (prcomp, R stats package v.4.0.3); the mean of the points corresponding to animals belonging to the same model (for each principal component) was then calculated to visualize each model as a separate point (the first two principal components are shown in Extended Data Figs. 1 and 2).

A hypergeometric test (base R package) was applied to evaluate the agreement between murine and human MASLD DEGs and DRPs. The test was performed on the results derived from the whole transcriptome as well as on those that were statistically significant and in the same direction of regulation as the human reference dataset hits (DEGs and DRPs; characterizing the ‘early disease development’, ‘all disease stages’ and ‘disease progression groups’). As a background, we used the whole transcriptome or the union of all the DEGs or DRPs (derived from these three groups; Fig. 4). For the human MASLD clinical features, an ANOVA test (base R package) was performed on the different MASLD groups for all the continuous variables (age, steatosis, inflammation, ballooning, fibrosis and NAS score). A chi-squared test was implemented to characterize the categorical variables (N, T2DM). Tukey’s test was then implemented to assess the post-hoc analysis significance by comparing the different MASLD groups (Supplementary Table 3).

A range of study design parameters from Supplementary Table 1 was evaluated in a PLSR model to examine their relationship with the MHPS (‘ability to induce MASH-fibrosis’ and ‘metabolic relevance’). Study parameters included both categorical (introduced as binary variables as follows: strain/background as C57BL/6J (yes or no) and C57BL/N (yes or no); main source of fat as lard (yes or no), Primex shortening hydrogenated (yes or no), coconut oil (yes or no), milkfat (yes or no) and palm oil (yes or no); WT/GA as genetically altered (yes or no); housing details as grouped housing (yes or no); sugar water? (yes or no); type of cage (IVC or standard); STZ and CCl_4_ together as chemicals (yes or no); details of the facility (SPF or standard); refined carbohydrates as sucrose in formula (yes or no) and sucrose and fructose in formula (yes or no)) and continuous variables (energy density (kcal g^−1^), carbohydrates (%kcal), fat (%kcal), proteins (%kcal), cholesterol (%), methionine (g kg^−1^), choline (g kg^−1^), duration experimental diet (weeks), room humidity and room temperature). The analysis focused on the larger subset of mouse models including HFD, HFDAMLD, WD, WD-STZ, WDAMLD or WDAMLD-CCl_4_ to enhance interpretability. The R package mixOmics v.2.8.0 (http://mixomics.org) was used to build the PLSR model and evaluate the optimal number of components. Relevant parameters were selected by considering the VIP (score threshold of 1). A visually modified version of the clustered image map from mixOmics was used to evaluate the correlations between the selected study design parameters and the MHPS.

Being a retrospective analysis, no statistical methods were used a priori to pre-determine sample sizes that depended on data availability from previous experiments. Therefore, some comparisons with low sample sizes might be exposed to type II error. Analyses including log_2_-transformed data met by default the normality assumptions. All other analyses were non-parametric and therefore normality testing was not a pre-requirement.

### Enrichment analysis

The FGSEA (https://github.com/ctlab/fgsea) package was used for fast pre-ranked gene set enrichment analysis of human and preclinical model analysis results. The analysis was applied to pathways from the KEGG database (v.2019)^56^.

### MHPS

We developed a scoring system termed MHPS that favors murine models that induce clinical features of MASLD (obesity, dyslipidemia, increased ALT and AST levels) and mimics the histopathological and pathophysiological characteristics of human MASLD. It provides a dual ranking score based on the metabolic significance of the models or their ability to induce MASH-fibrosis. Each of the two ‘arms’ of the MHPS scoring system is composed of three different layers (PHPS, HHPS and DHPS). Each layer provides a score that is added to generate the two arms (metabolic-relevant or fibrotic-relevant) of the final MHPS ranking (Fig. 1), determining the resemblance of the preclinical models to the human MASLD.

The PHPS comprises a seven-point scoring system, ranking the models against the human phenotypic outcomes based on BW, triglyceride and cholesterol levels, LW:BW ratio, and AST and ALT levels. Preclinical models closely mimicking human systemic metabolic disease and MASH characteristics are prioritized, while those showing a lower resemblance are penalized. Details of the PHPS scoring system are shown in Supplementary Table 5. Additionally, ALT and AST levels separating the animals into either no MASLD or MASH F2+ were evaluated by a receiver operating characteristic (ROC) curve analysis using Youden’s index to define the optimal threshold (Supplementary Fig. 3a,b). The final cutoff applied in the PHPS (Supplementary Table 5) calculation was defined as the combination of the optimal thresholds for ALT and AST (Supplementary Fig. 3c).

The HHPS assesses whether the histology samples mimic human MASLD/MASH (NAFLD/NASH) pathology and focuses on the key outputs, ranking the animals based on their ‘metabolic relevance’ or ‘ability to induce MASH-fibrosis’ as the main feature. HHPS includes some qualitative measures of human MASLD features that are expected in murine models to histologically mimic human MASH. Full details are shown in Supplementary Table 6.

The DHPS constitutes an adaptation of the DSEA and ‘gene2drug’ methods^57,58^, ranking the preclinical models against the human RNA-seq outcomes. DSEA provides an enrichment score that ranks the proximity of a specific preclinical model to a standard human reference dataset. This reference was constructed by focusing on reproducible transcriptional and pathway changes in human MASH based on the UCAM–VCU and EPoS datasets. This conservative approach addresses the limitations of both studies (for example, UCAM–VCU contains a limited control sample size and EPoS lacks lean controls) and provides confidence that the trend of regulation is similar between datasets, independently from possible power issues or biological differences in the cohorts. The DSEA method was applied in DEGs and differentially regulated KEGG pathways, after removing pathways not relevant for the liver and/or with redundant genes leading to their enrichment. To address the dual ranking system based on the metabolic or MASH-fibrosis relevance, human next-generation sequencing genes and pathways were divided into three groups: (1) all comparisons: hits homogeneously modulated at all disease stages (mild vs control, moderate vs mild, severe vs mild); (2) mild vs control: hits defining early disease stages (mild vs controls, but not moderate–severe vs mild) and (3) moderate–severe vs mild: hits defining progressive MASH (but not mild vs control). Hits from groups (1) and (2) or (1) and (3) were used as a reference for the metabolic-related or fibrotic-related ranking, respectively. This reference dataset in two layers has been used to rank the murine models based on their proximity to the expected outcome (how close their transcriptome changes are to human MASLD). The same layers of the analyses (genes or KEGG) performed in each murine model have been imputed in DSEA to complete the ranking. The outcome is an enrichment score and a *P* value that signifies how enriched the human disease signature is in the given model. A closer model to the expected phenotype has a higher enrichment score. To avoid bias in the interpretation of the results of DSEA, for all the non-statistically significant hits, the enrichment score is directly converted to zero, while enrichment scores from downregulated hits are multiplied by −1. The enrichment scores from the two different components of DSEA are then converted into a normalized enrichment score and averaged to generate the final DHPS. The basic principles of DSEA analysis that we have adapted to the needs of this study are described in Supplementary Fig. 4.

To merge the three different ranking methods described above (PHPS, DHPS, HHPS) into a final combined MHPS, the three components were scaled between 0 and 1 using the following formula:

$$Normalised\,scores=\frac{RowScore-\,\min (RowScore)}{\max (RowScore)-\,\min (RowScore)}$$

Each of these normalized scores contributed to one-third of the total score to generate the final MHPS score, which is directly translated into the final ranking.

### Visualization of results

Data tables were generated using Excel (Microsoft Office 2019). All analyses were performed in R (v.4.0.3). The heatmaps were produced with the package Pheatmap (v.1.0.12; https://rdrr.io/cran/pheatmap). For the visualization of the Sankey diagram (signal flow from the pathways towards the genes; Fig. 5), we used the function sankeyNetwork from the package networkD3 (v.0.4; https://rdocumentation.org/packages/networkD3/versions/0.4/topics/sankeyNetwork).

The functions ggscatter (https://rdocumentation.org/packages/ggpubr/versions/0.5.0/topics/ggscatter) and plot_grid (https://rdocumentation.org/packages/cowplot/versions/1.1.1/topics/plot_grid) from the packages ggpubr (v.0.4.0) and cowplot (v.1.1.1) were used for the scatterplots in Supplementary Fig. 1, adding the regression lines and calculating the Pearson correlation scores and the corresponding *P* values. Figs. 2, 6 and 7a and Extended Data Figs. 3 and 4 were produced using packages cowplot and ggplot2 (https://rdocumentation.org/packages/ggplot2/versions/3.4.0).

The receiver operating characteristic curves in Supplementary Fig. 3 were produced using the roc.curve function from the PRROC package (https://rdocumentation.org/packages/PRROC/versions/1.3.1/topics/roc.curve); sensitivity and specificity were calculated using the caret package (https://rdocumentation.org/packages/caret/versions/6.0-93). For visualization, the ggplot2 and ggpubr packages were applied.

### Reporting summary

Further information on research design is available in the Nature Portfolio Reporting Summary linked to this article.

### Supplementary information

Supplementary Information Supplementary Figs. 1–4 and legends.

Reporting Summary

Source Data Fig. S1 Log_2_FC values from the differential expression analysis and NES values from the KEGG pathway enrichment analysis, for all the statistically significant genes and pathways, respectively. These data are used to produce the four correlation plots in Fig. S1.

Supplementary Tables 1–8 Supplementary Tables S1, S2, S3, S4, S5, S6, S7, S8.

### Source data

Source Data Fig. 2 Phenotypic and histological processed data for each murine model including log_2_FC and *P* values (two-sided Mann–Whitney *U*-test) across BW, TGs, cholesterol, LW:BW% and ALT/AST, as well as the average score of steatosis, ballooning, inflammation, NAS and fibrosis.

Source Data Fig. 3 Processed data derived from the FGSEA (KEGG) pathway enrichment analysis, including tables with the NES, *P* values and adjusted *P* values.

Source Data Fig. 4 Processed data showing (1) the agreement between murine MASLD models and human data based on the list of significant DEGs and DRPs or the whole transcriptome and (2) corresponding *P* values of the hypergeometric test (one-sided) performed on the same comparison groups.

Source Data Fig. 5 Processed data showing the results from the differential expression analysis (selection of statistically significant DEGs enriching statistically significant modulated pathways) and the data producing the Sankey plot connecting those genes with the respective pathways and the different stages of MASLD development/progression. The DEGs matrix includes all the rodent models and the human comparisons (3-plets showing Log_2_FC, *P* values, adjusted *P* values) and the Sankey plot matrix includes all the nodes that have been used and information on the links (connections) between these nodes.

Source Data Fig. 6 Processed data representing the scores of PHPS, HHPS, DHPS and MHPS for the ranking according to ‘metabolic relevance’ or ‘ability to induce MASH-fibrosis’ respectively. Also included is BW log_2_FC and associated *P* value as well as average fibrosis score.

Source Data Fig. 7 Processed data of the PLSR analysis identifying the most influential study design parameters for the MHPS of ‘metabolic Relevance’ and ‘ability to induce MASH-fibrosis’. The data includes the VIP scores of the 1^st^ and 2^nd^ components and the correlations between study parameters and the two types of MHPS.

Source Data Fig. 8 Raw and processed data related to the selection of rodent models used to study the effects of treatments. (1) processed phenotypic data for the rodent models vs their corresponding controls (log_2_FC and *P* values) and histological scores (signed *P* values for steatosis, ballooning Inflammation fibrosis; two-sided Mann–Whitney *U*-test). (2) Processed data showing the results from the differential expression analysis of those models vs their corresponding controls. The DEGs matrix includes all the rodent models and the human comparisons (3-plets showing Log_2_FC, *P* values, adjusted *P* values). (4) Processed data derived from the FGSEA (KEGG) pathway enrichment analysis, including tables with the NES, *P* values and adjusted *P* values.

Source Data Extended Data Fig. 1 Processed data of the first 2 principal components of the PCA analysis, deriving from the batch-corrected gene expression data for the mouse models (and their corresponding controls). Data correspond to the results of PC1 and PC2 separately for all the animals as well as the average per animal model.

Source Data Extended Data Fig. 2 Processed data of the first 2 principal components of the PCA analysis, deriving from the batch-corrected gene expression data for the rat models (and their corresponding controls). Data correspond to the results of PC1 and PC2 separately for all the animals as well as the average per animal model.

Source Data Extended Data Fig. 3 Processed data representing the granular score components of the PHPS, HHPS and DHPS for each murine model according to the ‘metabolic relevance’ ranking scheme.

Source Data Extended Data Fig. 4 Processed data representing the granular score components of the PHPS, HHPS and DHPS for each murine model according to the ‘ability to induce MASH-fibrosis’ ranking scheme.

## Data Availability

All the murine data in this manuscript are original and unpublished except for 6J-WD-C0.2-32W (GSE110404)^59^, R-CDAA (GSE134715)^60^ and GAN-C2 REV/SEMA (GSE196908)^34^, which have been previously published and the raw data reused. Murine gene expression datasets have been deposited in the Array Express database (next-generation sequencing (NGS) accession number E-MTAB-12808; microarrays accession number E-MTAB-12817). All processed data used in or produced by this analysis have been deposited in Biostudies (accession number S-BSST1361; https://www.ebi.ac.uk/biostudies/studies/S-BSST1361), along with all murine metadata necessary for the interpretation, validation and expansion of the findings presented in this study. For those animals with available expression data, all metadata have also been deposited to the Array Express database. Human gene expression datasets and some metadata are publicly available (E-MTAB-9815, GSE130970, GSE135251); additional metadata are available upon request from the authors that originally published these datasets. Source data are provided with this paper.

## Code availability

R code and scripts are available upon request.
