# Supplementary material for: An unbiased ranking of murine dietary models based on their proximity to human metabolic dysfunction-associated steatotic liver disease (MASLD)
Source: Nat Metab. 2024 Jun 12;6(6):1178–96. doi: 10.1038/s42255-024-01043-6 (PMC11199145; doi:10.1038/s42255-024-01043-6)
Supplement: Supplementary file 1 — Supplementary Figs. 1–4 and legends. [file 42255_2024_1043_MOESM1_ESM.pdf]

nature metabolism

Supplementary information https://doi.org/10.1038/s42255-024-01043-6

An unbiased ranking of murine dietary models based on their proximity to human metabolic dysfunction-associated steatotic liver disease (MASLD)

In the format provided by the authors and unedited

LEGENDS TO SUPPLEMENTARY FIGURES:

Figure S1. Reproducibility of the human transcriptomic data between datasets

Correlation of the DEGs (A-B; Log2FC) or DRPs (C-D; NES) between the two human datasets (UCAM/VCU and EPoS) when considering the different comparisons ("moderate vs mild" or "severe vs mild"). These plots depict only the hits included in the analyses following the filtering strategy described in the methods. The R score for each plot represents the Pearson correlation scores, followed by the corresponding p-value; the null hypothesis (two-sided statistical test) states that there is no linear relationship between the two correlated variables. The grey bands around the four regression lines denote the 95% confidence interval. *The minimum p-value when performing this test in R was set by default to 2.2e-16.*

Figure S2. Integrated Transcriptomic Analysis Pipeline

The pipeline has been used to analyse the human UCAM/VCU and EPoS RNASeq data (human reference datasets) and the murine RNASeq/Microarray data (details in the methods section). Abbreviations: CEL files; Files created by DNA microarray image analysis software, CPM; Counts per million, COMBAT; Batch effect correction tool, DESeq2; Differential expression analysis based on the Negative Binomial (Gamma-Poisson) distribution, FGSEA; Fast gene set enrichment analysis, Hisat2; Graph-based alignment tool for sequencing reads to the reference genome, HTSeq; High-throughput sequence analysis, Limma; Linear models for microarray data, QC; Quality Control, RMA; Robust Multichip Average.

Figure S3: Thresholds to establish hypertransaminasemia in rodents have been optimised against histological outcomes

Receiver operating characteristic (ROC) curves with Youden's index have been used to define sufficiently sensible/specific cut-offs in rodents for AST/ALT when attempting to predict histological outcomes (presence of MASH with significant fibrosis). In murine models, ALT (A) and AST (B) appeared to be highly accurate in predicting MASH-Fibrosis; moreover, using a combined threshold of the two, the accuracy improved (C). This approach was implemented in the PHPS following the strategy described in Table S5.

Figure S4. The DSEA Human Proximity Score (DHPS)

After DEG and DRP processing analysis of murine and human datasets (see methods), the Drug Set Enrichment analysis DSEA) tool (https://dsea.tigem.it/) was used to rank the models based on a reference dataset as previously described [35,36]. This ranking was independently repeated for both DEG and DRP, and the enrichment score (ES)

was converted into a normalised ES (NES), and results were averaged after normalisation to generate the final DHPS. Interpretation: the closer DHPS is to “1”, the more the murine data are aligned to the Human Reference Dataset.

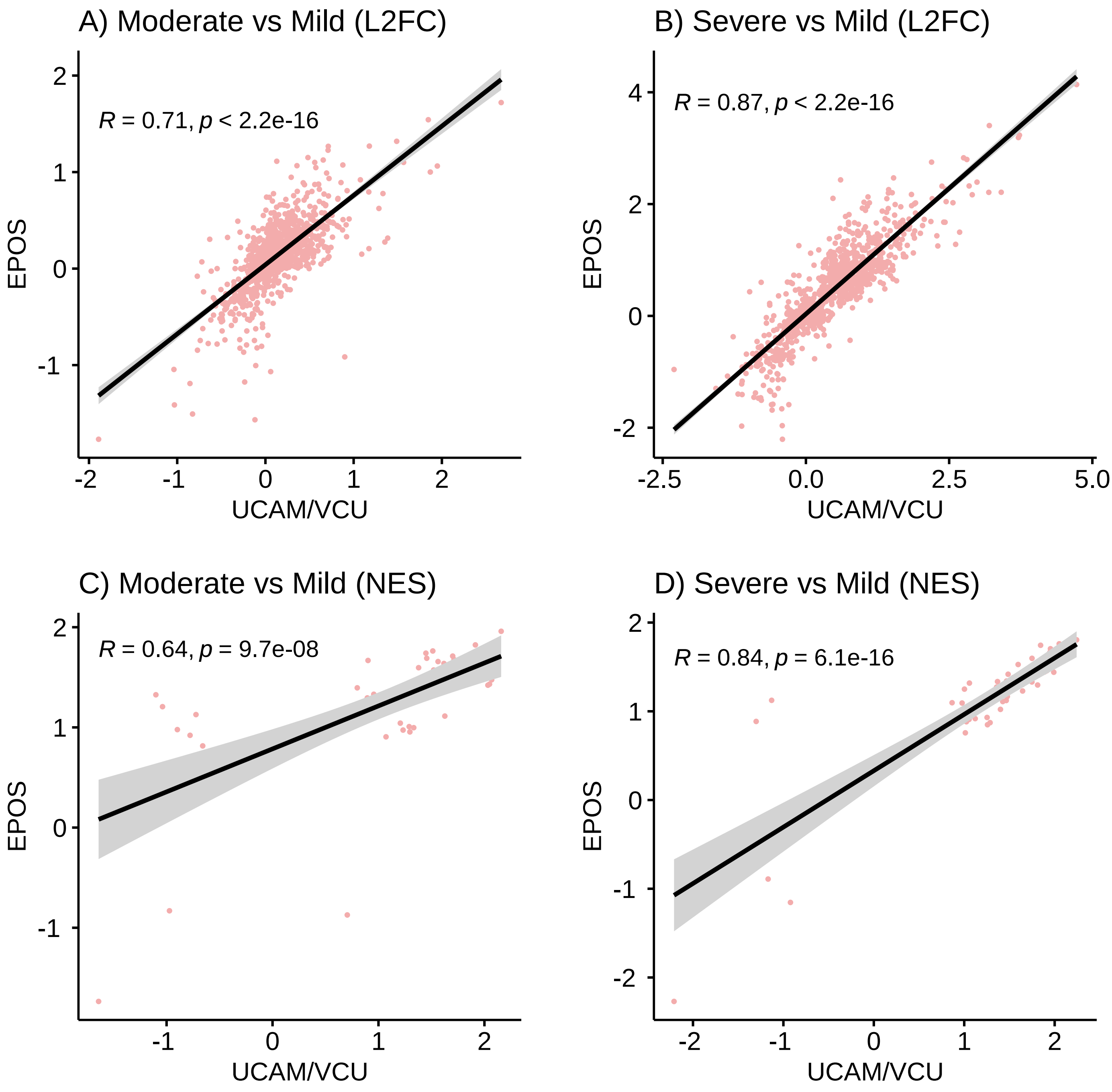
A) Moderate vs Mild (L2FC)
R = 0.71, p < 2.2e-16
EPOS
UCAM/VCU
B) Severe vs Mild (L2FC)
R = 0.87, p < 2.2e-16
EPOS
UCAM/VCU
C) Moderate vs Mild (NES)
R = 0.64, p = 9.7e-08
EPOS
UCAM/VCU
D) Severe vs Mild (NES)
R = 0.84, p = 6.1e-16
EPOS
UCAM/VCU

Figure S1

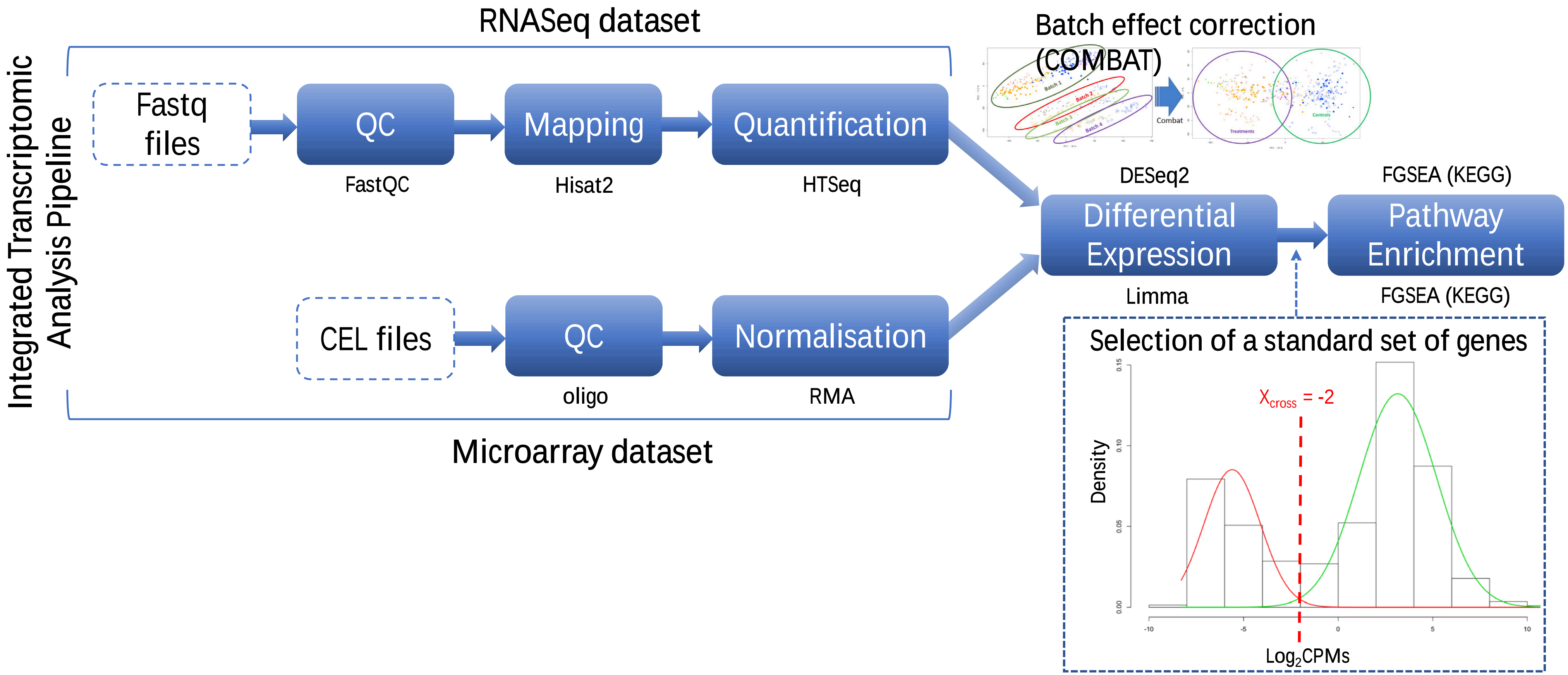
RNASeq dataset
Integrated Transcriptomic Analysis Pipeline
Fastq files
QC
FastQC
Mapping
Hisat2
Quantification
HTSeq
CEL files
QC
oligo
Normalisation
RMA
Microarray dataset
Batch effect correction (COMBAT)
Combat
DESeq2
Differential Expression
Limma
FGSEA (KEGG)
Pathway Enrichment
FGSEA (KEGG)
Selection of a standard set of genes
Xcross = -2
Density
Log2CPMs

Figure S2

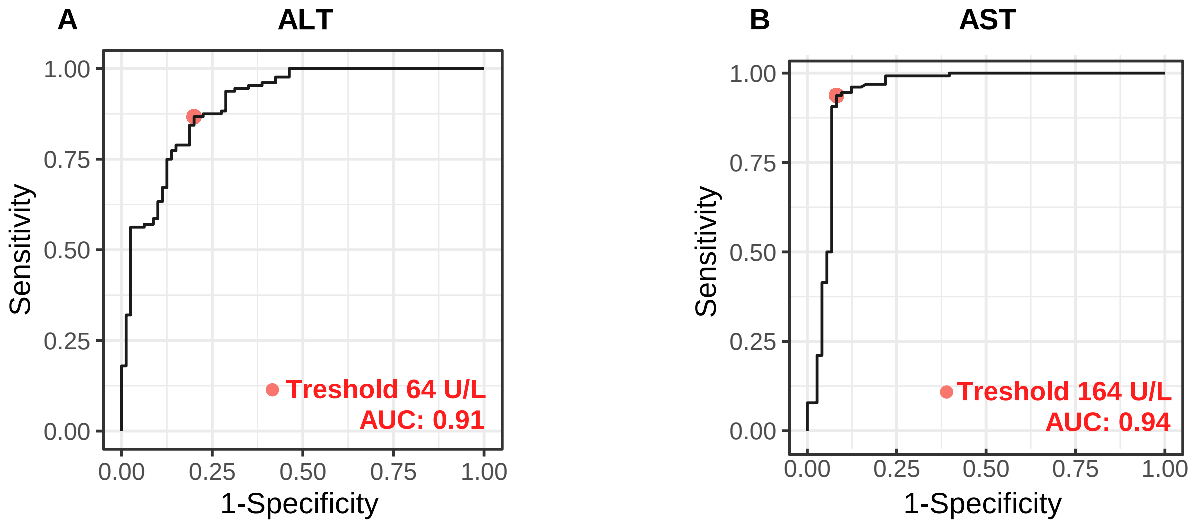

C

Cutoff	SPECIFICITY	SENSITIVITY
ALT (64)	0.78	0.87
AST (164)	0.92	0.94
ALT (64) & AST (164)	0.93	0.83

Figure S3

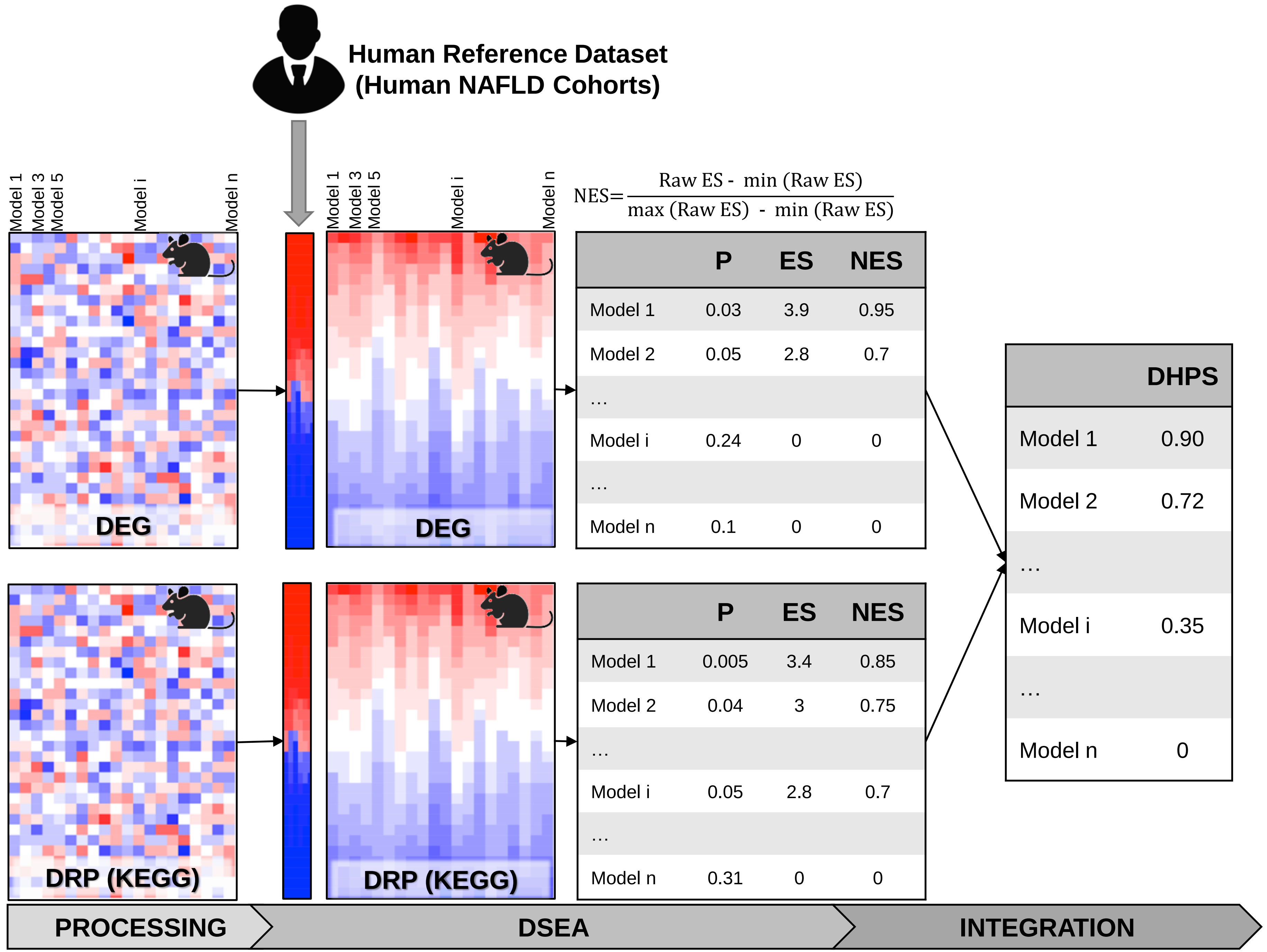
Human Reference Dataset
(Human NAFLD Cohorts)
Model 1
Model 3
Model 5
Model i
Model n
NES= Raw ES - min (Raw ES) / max (Raw ES) - min (Raw ES)
DEG
DEG
P ES NES
Model 1 0.03 3.9 0.95
Model 2 0.05 2.8 0.7
…
Model i 0.24 0 0
…
Model n 0.1 0 0
DRP (KEGG)
DRP (KEGG)
P ES NES
Model 1 0.005 3.4 0.85
Model 2 0.04 3 0.75
…
Model i 0.05 2.8 0.7
…
Model n 0.31 0 0
DHPS
Model 1 0.90
Model 2 0.72
…
Model i 0.35
…
Model n 0
PROCESSING
DSEA
INTEGRATION

Figure S4
