# Supplementary material for: An unbiased ranking of murine dietary models based on their proximity to human metabolic dysfunction-associated steatotic liver disease (MASLD)
Source: Nat Metab. 2024 Jun 12;6(6):1178–96. doi: 10.1038/s42255-024-01043-6 (PMC11199145; doi:10.1038/s42255-024-01043-6)
Supplement: Supplementary file 2 — Reporting Summary [file 42255_2024_1043_MOESM2_ESM.pdf]

nature portfolio

Corresponding author(s): Michele Vacca, Dina Tiniakos, James W Perfield, Evangelia Petsalaki, Peter Davidsen, Antonio Vidal-Puig

Last updated by author(s): Mar 22, 2024

Reporting Summary

Nature Portfolio wishes to improve the reproducibility of the work that we publish. This form provides structure for consistency and transparency in reporting. For further information on Nature Portfolio policies, see our Editorial Policies and the Editorial Policy Checklist.

Statistics

For all statistical analyses, confirm that the following items are present in the figure legend, table legend, main text, or Methods section.

n/a	Confirmed	
☐	☒	The exact sample size (*n*) for each experimental group/condition, given as a discrete number and unit of measurement
☐	☒	A statement on whether measurements were taken from distinct samples or whether the same sample was measured repeatedly
☐	☒	The statistical test(s) used AND whether they are one- or two-sided<br>*Only common tests should be described solely by name; describe more complex techniques in the Methods section.*
☐	☒	A description of all covariates tested
☐	☒	A description of any assumptions or corrections, such as tests of normality and adjustment for multiple comparisons
☐	☒	A full description of the statistical parameters including central tendency (e.g. means) or other basic estimates (e.g. regression coefficient) AND variation (e.g. standard deviation) or associated estimates of uncertainty (e.g. confidence intervals)
☐	☒	For null hypothesis testing, the test statistic (e.g. *F*, *t*, *r*) with confidence intervals, effect sizes, degrees of freedom and *P* value noted<br>*Give P values as exact values whenever suitable.*
☒	☐	For Bayesian analysis, information on the choice of priors and Markov chain Monte Carlo settings
☒	☐	For hierarchical and complex designs, identification of the appropriate level for tests and full reporting of outcomes
☐	☒	Estimates of effect sizes (e.g. Cohen's *d*, Pearson's *r*), indicating how they were calculated

Our web collection on statistics for biologists contains articles on many of the points above.

Software and code

Policy information about availability of computer code

Data collection	Microsoft Excel – Microsoft Office 2019
Data analysis	RStudio Inc, The R Foundation for Statistical Computing – v4.0.3;<br>For NGS data analysis we used: FastQC - v0.11.9; https://github.com/s-andrews/FastQC, Hisat2 - v2.1.0, HTSeq - v0.11.1, biomaRt – v2.54.0, qnorm() - standard R function, Limma - v3.38.2, COMBAT - sva package -v3.38.0, DESeq2 – v1.26.0, Benjamini-Hochberg method – FDR calculation, cpm – edgeR -v3.32.1, FGSEA - https://github.com/ctlab/fgsea, KEGG database – v2019.<br>For the phenotype and histology raw p-value calculation we used the Mann-Whitney U test (wilcox.test – stats v3.6.2). For the hypergeometric test we used the function phyper (base R package; REF: Johnson, N. L., Kotz, S., and Kemp, A. W. (1992) Univariate Discrete Distributions, Second Edition. New York: Wiley). For the human clinical feature characterisation we used ANOVA (base R package; for the continuous variables) and chi-squared test (for the chategorical variables). The function prcomp was used for the estimations of the principal components in the pca plots (package stats - v4.0.3).<br>For the visualisation of the results we used the following tools: The heatmaps were produced with the package "Pheatmap" (v.1.0.12; https://rdrr.io/cran/pheatmap). The Sankey diagram was produced with the function "sankeyNetwork" from the package "networkD3" (v.0.4; https://rdocumentation.org/packages/networkD3/versions/0.4/topics/sankeyNetwork). The functions "ggscatter" (https://rdocumentation.org/packages/ggpubr/versions/0.5.0/topics/ggscatter) and "plot_grid" (https://rdocumentation.org/packages/cowplot/versions/1.1.1/topics/plot_grid) from the packages "ggpubr" (-v0.4.0) and "cowplot" (-v1.1.1), were used for the scatter plots, adding the regression lines, and calculating the R (Pearson correlation) scores and the corresponding p-values. The ROC-curves were produced using the "roc.curve" function from the "PRROC" package (https://rdocumentation.org/packages/PRROC/versions/1.3.1/topics/roc.curve), while sensitivity and specificity were calculated using the "caret" package (https://rdocumentation.org/packages/caret/versions/6.0-93). For visualisation, the ggplot2 (https://rdocumentation.org/packages/ggplot2/versions/3.4.0) and ggpubr packages were applied. The package ggplot was used for the visualisation of the pca plots. The partial least squares regression was performed using the "pls" function from the package

"mixOmics" (http://mixomics.org/, v2.8.0), while VIP scores and correlation structure between the study design and MHPS were extracted using the functions "vip" and "cim" functions respectively. The correlation structure result from cim was visualised using "Pheatmap" as described above.
Code is available upon request.

For manuscripts utilizing custom algorithms or software that are central to the research but not yet described in published literature, software must be made available to editors and reviewers. We strongly encourage code deposition in a community repository (e.g. GitHub). See the Nature Portfolio guidelines for submitting code & software for further information.

Data

Policy information about availability of data

All manuscripts must include a data availability statement. This statement should provide the following information, where applicable:

- Accession codes, unique identifiers, or web links for publicly available datasets
- A description of any restrictions on data availability
- For clinical datasets or third party data, please ensure that the statement adheres to our policy

All the murine data in this manuscript are original and unpublished, except for the 6J-WD-C0.2-32W (GSE110404)49, R-CDAA (GSE134715)50, and GAN-C2 REV/SEMA (GSE196908)34 that have been previously published, and raw data reused. Murine Gene Expression datasets have been deposited in the Array Express database (NGS accession number: E-MTAB-12808; Microarrays accession number: E-MTAB-12817). All processed data used in or produced by this analysis have been deposited in Biostudies (Accession number: S-BSST1361, https://doi.org/10.6019/S-BSST1361), along with all murine metadata necessary for the interpretation, validation, and expansion of the findings presented in this study. For those animals with available expression data, all metadata have also been deposited to the Array Express database. Human Gene Expression datasets and some metadata are publicly available (E-MTAB-9815, GSE130970, GSE135251); additional metadata are available upon request from the authors that originally published these datasets.

Human research participants

Policy information about studies involving human research participants and Sex and Gender in Research.

Reporting on sex and gender	The human data (publicly available datasets) have been generated in males and females (sex). Gender information was not available to the investigators.
Population characteristics	The UCAM (University of Cambridge) / VCU (Virginia Commonwealth University) super-cohort consisted of 2 publicly available (E-MTAB-9815, GSE130970) datasets (TOTAL: 136 Patients) previously described by our teams. All the patients had a clinical diagnosis of NAFLD and histology scores according to the NASH CRN Scoring System. Patients were divided into CTRL (n = 4) and NAFLD (n = 132) sub-clustered against fibrosis (Mild i.e. F0: n = 52; Moderate i.e. F1-2: n = 50; Severe i.e. F3-4: n = 30).<br><br>The EPoS (Newcastle University) Cohort is a large cohort of NAFLD patients with NGS available (GSE135251) recruited in different EU institutions and previously described by our teams. All the patients had a clinical diagnosis of NAFLD, and histology was centrally scored according to the NASH CRN Scoring System as previously described; 38 patients of the initial cohort were removed as they overlapped with the UCAM/VCU dataset. The remaining 168 NAFLD patients were sub-clustered against fibrosis (Mild i.e. F0: n = 47; Moderate i.e. F1-2: n = 64; Severe F3-4: n = 57).<br>The covariate-relevant population characteristics included the gender for datasets E-MTAB-9815 and GSE130970 (publicly available), and the gender and site for dataset GSE135251 (requested and confidentially provided by the authors of the original study by email).
Recruitment	The Human data have been produced starting from already described studies with NGS data already available. The description of the recruitment was provided in the original publications (PMID: 33722690, 31467298, 33268509).
Ethics oversight	The relevant Ethics Committees (UCAM/VCU Cohort: East of England Research Ethics Committee, Virginia Commonwealth University; EPoS Cohort: multiple Ethical Committees in the participating countries) approved these studies as detailed in the original publications. The description of the relevant ethical approvals for this study has been provided in the original publications (PMID: 33722690, 31467298, 33268509).

Note that full information on the approval of the study protocol must also be provided in the manuscript.

Field-specific reporting

Please select the one below that is the best fit for your research. If you are not sure, read the appropriate sections before making your selection.

☒ Life sciences ☐ Behavioural & social sciences ☐ Ecological, evolutionary & environmental sciences

For a reference copy of the document with all sections, see nature.com/documents/nr-reporting-summary-flat.pdf

Life sciences study design

All studies must disclose on these points even when the disclosure is negative.

Sample size	Given the retrospective study design, statistical analyses were performed on the basis of data/sample availability. Power analysis has therefore not been performed.
Data exclusions	Animal models without centralised assessed histology, transcriptomics data, and phenotypic information were not included in the analyses. Transcriptomics data: For microarrays, technical outliers with only 1 or 2 samples processed for a given scan date were removed, retaining 13-15 samples for each timepoint/diet. RNA-seq inclusion criteria were the following: GC% content was approximately 50%, more than 10 million reads passed the quality filtering, and more than 80% of the reads per sample were mapped to the reference genome (all samples passed the QC).
Replication	Human data used to compare murine data were based on highly reproducible hits that resulted significant and with the same direction of modulation in the two datasets studied therefore ensuring high replicability among datasets. Each murine experiment was repeated once.
Randomization	Given the retrospective study design, randomisation is not applicable to this study.
Blinding	Given the retrospective study design, the blinding of the investigators during data acquisition is not applicable to this study. For the histology, both the histology team at IBBL and pathologists were blind when acquiring/assessing control/treatment diets. They were also unaware about the results of the other data layers not to influence their judgment.

Reporting for specific materials, systems and methods

We require information from authors about some types of materials, experimental systems and methods used in many studies. Here, indicate whether each material, system or method listed is relevant to your study. If you are not sure if a list item applies to your research, read the appropriate section before selecting a response.

Materials & experimental systems

n/a	Involved in the study	
☒	☐	Antibodies
☒	☐	Eukaryotic cell lines
☒	☐	Palaeontology and archaeology
☐	☒	Animals and other organisms
☒	☐	Clinical data
☒	☐	Dual use research of concern

Methods

n/a	Involved in the study	
☒	☐	ChIP-seq
☒	☐	Flow cytometry
☒	☐	MRI-based neuroimaging

Animals and other research organisms

Policy information about studies involving animals; ARRIVE guidelines recommended for reporting animal research, and Sex and Gender in Research

Laboratory animals	Mouse: C57BL/6J, C57BL/6JRj, C57BL/N, C57B/6 S129J, C57BL/6J-LDLRKO, C57BL/6J-LEPTIN-KO, C57BL/6J-MC4RKO, db/db, ob/ob.<br>Rat: RjHan:Wi, ZSF1-Leprfa Leprcp/Crl.<br>Specific details for each individual model are included in Table S1 and Table S7
Wild animals	No wild animals were used in the study.
Reporting on sex	Experiments were mainly performed in Male rodents (21 F; 649 M). Sex information for each model is described in Tables S1 and S7.
Field-collected samples	No field collected samples were used in the study.
Ethics oversight	Relevant animal welfare authorities approved all the animal experiments that complied with national/international guidelines.

Note that full information on the approval of the study protocol must also be provided in the manuscript.
